# Alzheimer’s Disease polygenic risk, the plasma proteome, and dementia incidence among UK older adults

**DOI:** 10.1007/s11357-024-01413-8

**Published:** 2024-11-26

**Authors:** May A. Beydoun, Hind A. Beydoun, Zhiguang Li, Yi-Han Hu, Nicole Noren Hooten, Jun Ding, Sharmin Hossain, Christian A. Maino Vieytes, Lenore J. Launer, Michele K. Evans, Alan B. Zonderman

**Affiliations:** 1 Laboratory of Epidemiology and Population Sciences, National Institute On Aging, NIA/NIH/IRP, NIH Biomedical Research Center, National Institute On Aging Intramural Research Program, 251 Bayview Blvd, Suite 100, Baltimore, MD 21224 USA; 2 VA National Center On Homelessness Among Veterans, U.S. Department of Veterans Affairs, Washington, DC 20420 USA; 3 Department of Management, Policy, and Community Health, School of Public Health, University of Texas Health Science Center at Houston, Houston, TX 77030 USA; 4 Translational Gerontology Branch, National Institute On Aging, NIA/NIH/IRP, Baltimore, MD 21224 USA; 5 Department of Human Services (DHS), State of Maryland, Baltimore, MD 21202 USA

**Keywords:** Alzheimer’s Disease polygenic risk, Plasma proteomic biomarkers, Dementia, Aging

## Abstract

Alzheimer’s Disease (AD) is a complex polygenic neurodegenerative disorder. Its genetic risk’s relationship with all-cause dementia may be influenced by the plasma proteome. Up to 40,139 UK Biobank participants aged ≥ 50y at baseline assessment (2006–2010) were followed-up for ≤ 15 y for dementia incidence. Plasma proteomics were performed on a sub-sample of UK Biobank participants (*k* = 1,463 plasma proteins). AD polygenic risk scores (PRS) were used as the primary exposure and Cox proportional hazards models were conducted to examine the AD PRS-dementia relationship. A four-way decomposition model then partitioned the total effect (TE) of AD PRS on dementia into an effect due to mediation only, an effect due to interaction only, neither or both. The study found that AD PRS tertiles significantly increased the risk for all-cause dementia, particularly among women. The study specifically found that AD PRS was associated with a 79% higher risk for all-cause dementia for each unit increase (HR = 1.79, 95% CI: 1.70–1.87, *P* < 0.001). Eighty-six plasma proteins were significantly predicted by AD PRS, including a positive association with PLA2G7, BRK1, the glial acidic fibrillary protein (GFAP), neurofilament light chain (NfL), and negative with TREM2. Both GFAP and NfL significantly interacted synergistically with AD PRS to increase all-dementia risk (> 10% of TE is pure interaction), while GFAP was also an important consistent mediator in the AD PRS-dementia relationship. In summary, we detected significant interactions of NfL and GFAP with AD PRS, in relation to dementia incidence, suggesting potential for personalized dementia prevention and management.

**Supplementary Information:**

The online version contains supplementary material available at 10.1007/s11357-024-01413-8.

## Introduction

Dementia is described as loss of global abilities in several cognitive domains and inability to carry out daily living activities [1–3]. The estimated prevalence of dementia among those over 60y of age is 4.7% [2–4], with 4.6–7.7 million new cases added each year globally (3.5–10.5 per 1,000) [2–7]. Alzheimer’s disease (AD) is the most common dementia sub-type, accounting for 60–80% of cases [2–4] and the leading cause of old age disability. AD is a progressive neurodegenerative disorder with complex etiology that begins with episodic memory decline and leads to impairment in other cognitive domains [2, 3, 8].

Given the lack of cure for dementia, prevention is essential. Therefore, research endeavors have focused on modifiable and non-modifiable risk or protective factors associated with dementia between mid-life and older age. In fact, recently, the 2020 *Lancet commission* and its 2024 update concluded that 40% of dementia risk can be attributed to potentially modifiable risk factors, each being more effective at predicting this outcome, either at early-, mid-, or later-life [2, 3, 9, 10]. Nevertheless, a significant part of dementia risk can be explained by genetic factors, including the APOE ε4 allele dosage which is highly predictive of sporadic AD [2, 9].

Complex conditions, such as health outcomes or behaviors, are often highly polygenic, suggesting an influence of multiple gene variations; consequently, a single nucleotide polymorphism (SNP) is likely insufficient to capture this level of complexity. A polygenic risk score (PRS) combines thousands of individual genetic loci across the human genome, weighing them according to effect sizes derived from a genome-wide association study. This results in a single quantitative measurement of genetic risk for a specific trait, increasing the power of genetic analyses. PRSs are quite specific to the phenotypes they predict, including AD. Previous studies have focused on potential interactions between AD PRS levels and modifiable risk factors, such as measures of cardiovascular health, in relation to dementia or AD risk (e.g., [11]). Furthermore, evidence was found of sex differentials in the relationships between APOE ε4 allele dosage, particularly in its association with various dementia sub-types, including incidence of all-cause dementia or AD [12–14]. Elements of the plasma proteome and associated pathways may be proximal causal or predictive factors behind dementia risk. This proteome can possibly mediate or moderate the association between AD PRS and all-cause dementia. Nevertheless, most research pertaining to AD-related gene polymorphisms or AD PRS’s link to the plasma proteome, with few exceptions (e.g., [15]), has targeted few proteins. In contrast, both focused and large-scale proteomics have linked the plasma proteome to all-cause dementia [16–21].

We used data from the UK Biobank to assess sex-specific relationship between AD PRS and incidence of all-cause dementia in this retrospective cohort study. We attempted to explain apparent relationships through the plasma proteome, given the attention paid by most studies on small cohort sizes or a limited number of proteins. Differential mediation and moderation among men and women were also tested in parts of the analyses.

## Materials and methods

### Database

The UK Biobank is a population-centric research initiative designed to enhance illness prevention, diagnosis, and treatment [22]. The study collects data from more than 500,000 middle-aged and older persons in the UK from 2006 to 2010, including insights into demographic characteristics, socioeconomic position, lifestyle factors, medical history, genetic data, metabolomic and proteomic profiles, and neuroimaging markers [22]. The data is worldwide accessible through the UK Biobank Research Analysis Platform (UKB RAP) [22]. The Northwest Multi-Centre Research Ethics Committee approved the UK Biobank project, while the Institutional Review Board of the National Institutes of Health and the UK Biobank Access Management System have approved the current project under application #77,963.

### Dementia outcomes

Dementia occurrence data was generated using a specific algorithm (See fields 42,018 and 42,020). Using these data, we excluded participants whose age at dementia onset was less than their respective baseline assessment ages [23]. ICD-10 codes of F00 or G30 were utilized for the purpose of identifying the AD sub-type of dementia. Nevertheless, dementia of any cause used several codes (A81.0, F00, F01, F02, F03, F05, G30, G31.0, G31.1, G31.8, and I67.3). Consequently, the date of first episode of any dementia was determined by selecting the earliest date linked to this outcome of interest. In a similar fashion, AD incidence was determined but restricting ICD-10 codes to F00 or G30.

### AD polygenic risk score

PRS scores were developed and applied to meta-analyzed (and, where possible, ancestry specific) GWAS summary statistics that were either completely extracted from external GWAS data (the Standard PRS set) or from a combination of external and internal UK Biobank data (the Enhanced PRS set) using a Bayesian approach[24], found in: https://www.medrxiv.org/content/10.1101/2022.06.16.22276246v1. Here, only the Standard PRS was utilized. DNA samples from UK Biobank subjects who were genotyped using a custom Axiom genotyping array assaying 825,927 genetic variations were utilized to construct the standard PRS for AD. Genome-wide imputation was then performed. In an earlier study, the genotyping platform utilized in UK Biobank was detailed [25]. The UK Biobank-released Standard PRS for AD was created using a meta-analysis of solely external GWAS data (not including subjects from UK Biobank or UKB-free). Next, PRS for AD was standardized into the distribution of approximately zero mean and unit variance in each ancestry group [24]. After evaluating PRS for AD (Field ID = 26,206), we mainly used them as continuous variables, while stratifying them into tertiles in part of the analysis [24] (Appendix II, OSM 1). Furthermore, an alternative AD PRS was also generated from raw genome imputation data in *bgen* format using a standard pipeline described elsewhere and was used for replication purposes, also relying on GWAS results that were only partly external to UKB [26] (Appendix II, OSM 5).

As a sensitivity analysis, both AD PRS scores (main and secondary) were correlated with each other and compared across levels of ApoE4 carrier status [27]. Specifically, two genetic variants, rs429358 and rs7412, were used to directly genotype and define the APOE haplotypes (ε2/ε3/ε4). Individuals classified as APOE ε4 carriers had one or two ε4 alleles; those without such alleles were considered APOE ε4 noncarriers (e.g., [27]). Quality control measures in this previous study [27] were comparable to those applied to genetic principal components, excluding among others participants with strong kinship.

### OLINK proteomics

Of the initial ~ 500,000 UK Biobank participants, 54,306 were selected for further analyses of their plasma samples using the Olink® Explore 1536 Proteomics platform, relying on sample collected at baseline assessment. The present study uses data on 1,463 unique proteins that cover inflammation, cancer, cardiometabolic and neurological panels. Normalized protein expression (NPX) through Log2 transformation is used to represent this plasma protein data. For more details, see Sun et. al [28] and OSM 1 within Appendix II.

### Covariates

Covariates included age, sex, squared-age to allow for non-linear age effect, and the first 20 genetic principal components (https://www.nealelab.is/uk-biobank/ukbround2announcement. ), (Appendix II, OSM 1).

### Study sample selection

The UK Biobank sample included 502,268 adults, with 384,548 aged ≥ 50 years at baseline. There were 372,434 participants with complete data on socio-demographic, AD PRS, and genetic principal component factors. About 10% had proteomic data, leaving 40,188 people with full plasma protein data. After eliminating 49 prevalent dementia patients, the final sample size was 40,139, with 1,167 having incident all-cause dementia for > 15 years (Appendix III, Figure S1).

### Statistical methods

Stata 18.0 (StataCorp, College Station, TX) was utilized for the key components of this analysis. Means and proportions were estimated in the entire sample with additional stratification by sex and comparison by this demographic factor accomplished using several bivariate linear and multinomial logit models.

At a second stage, exposure-outcome relationships were assessed using a time-to-event approach, with time defined as the interval between age at entry into the study (≥ 50 years) and age at exit. The latter could be either age of incident outcome or age at censoring (death or end of follow-up by October 31st, 2021). To show differences in dementia-free survival probability, Kaplan–Meier survival rates were computed and compared using a log-rank test across the tertile form of the exposure of interest, AD PRS: T_1_ (low AD PRS), T_2_ (medium AD PRS), or T_3_ (high AD PRS). We then constructed Cox proportional hazards (PH) models, which aimed at examining the association between AD PRS (in tertile format) and incidence of dementia, with further adjustment for baseline age, squared age, gender, and the first 20 genetic major components.

Third, AD PRS was subsequently entered as a main predictor in a series of multiple linear regression models. These models had each of the proteomic mediator as an alternative outcome, among the 1,463 plasma proteins. The model simultaneously adjusted for potential confounders. Stata parmby, qqval and multproc commands were used, and a volcano plot was generated using R ggplot package, which clear visualizes *p*-values and effect sizes for each of the 1,463 equations along with those that passed multiple testing using Bonferroni criteria for adjustment (Type I error reduced to 0.05/1,463). The top hits were chosen by setting effect sizes to > 0.20 in absolute value (corresponding to 1/5 SD higher plasma protein per SD of AD PRS), a value known as a cutoff between very weak (or very small) and weak (or small) effect size when considering the association between two continuous variables, using several standards, including Cohen's D (https://cran.r-project.org/web/packages/effectsize/vignettes/interpret.html). This step was only included if the Bonferroni correction identified > 500 candidate mediators.

Fourth, each of the plasma proteins were then entered into a four-way decomposition model for the total effect of AD PRS on incidence of dementia, as alternative potential mediators and moderator. The final equation in this model was a Cox PH model. OSM3 in Appendix II and Appendix IV delves deeper into four-way decomposition. All models in the 4-way decomposition method accounted for previously mentioned covariates. Tabular data coupled with a heatmap are used to visualize findings for selected key mediators that passed Bonferroni correction and other selection criteria above. Results were categorized as “no mediation,” “consistent” (controlled direct effect (CDE) < total effect (TE) in absolute value, sign of pure indirect effect (PIE) is the same as for CDE), and “inconsistent mediation” (CDE > TE in absolute value, PIE and CDE have opposing signs) [29–33]. Key identified mediators or moderators of the AD PRS-dementia association are highlighted in terms of their function and relationship with AD PRS and dementia in prior studies.

The screened plasma proteins with significant PIE at 0.05 type I error were included in a principal components analysis (PCA), a method of dimension reduction, whenever TE was found to be statistically significant at a type I error of 0.05, along with a consistent mediation (CDE < TE in absolute value, and PIE has the same sign as CDE) (OSM4, Appendix II). In the event that several principal components were extracted, orthogonal rotation was implemented so as to obtain a simple structure and for ease of interpretation. The Kaiser rule (eigenvalue greater than one) was used to specify the number of extracted components. These PCA scores were predicted using the regression technique and then included into another set of 4-way decomposition models to evaluate the degree of mediation and/or moderation by these components in the TE of AD PRS on dementia incidence, while adjusting for earlier mentioned exogenous variables. In most analyses, sex was the primary stratifying factor.

OLINK insight pathways and STRING analyses were also performed as a secondary analysis on all mediators detected from four-way decomposition of the proteome (i.e., *k* = 1,463 proteins) in the relationship between AD PRS and dementia incidence (Appendix II OSM 7; Appendix VIII (Figure S4); Appendix X). In another secondary analyses, top selected plasma proteins and generated principal components for consistent mediators obtained from the 2019 IGAP AD PRS (main analysis) were tested as potential mediators and/or moderators between a newer version of AD PRS (2022 GWAS) and all-cause dementia [26]. This version is the newest among three large GWAS studies on AD over the past 5 years [26, 34, 35]. Details regarding data processing used to obtain this new version of AD PRS are found in OSM5 (Appendix II). Main and secondary analyses were compared for the purpose of replication. Two other secondary analyses were also carried out, whereby four-way decomposition was carried out on both the main and new AD PRS scores [24, 26] with outcome being incident AD, after screening for the plasma proteins that were most strongly associated with the main AD PRS of interest. The second analysis correlated the two AD PRS scores [24, 26] and compared their means across APOE4 carrier status. This analysis also assessed the relationship between the new AD PRS and all-cause dementia as well as AD incidence. Output for these secondary analyses is provided on github: https://github.com/baydounm/UKB-paper12-supplementarydata.

### Data availability statement

UK Biobank is a large-scale biological database and research resource containing detailed genetic and health information from > 500,000 UK individuals. The database is frequently updated and is available to approved researchers worldwide. Access to these datasets should be requested through https://www.ukbiobank.ac.uk/.

## Results

**Fig. 1:**
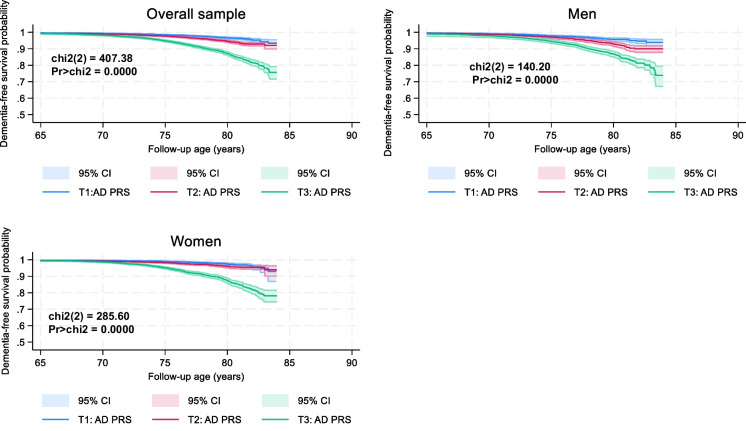
Alzheimer’s Disease polygenic risk tertiles vs. all-cause dementia, overall and by sex: UK biobank 2006–2021. *Abbreviations*: chi2 = chi-square test; CI = confidence interval; KM = Kaplan Meier; LE8 = Life’s Essential 8; Pr = *P*-values; T1 = First tertile (lowest AD polygenic risk); T2 = Second teritle (medium AD polygenic risk); T3 = Uppermost tertile (Highest AD polygenic risk); UK = United Kingdom. *Note*: Chi2 refers to a log-rank test. Details are provided on github: https://github.com/baydounm/UKB-paper12-supplementarydata

There were few sex differences across variables of interest, mainly age, and few genetic principal components (Table 1). Nevertheless, AD PRS mean and tertile distribution was homogeneous between men and women, while all-cause dementia cumulative risk was greater among men. Conversely, men and women had comparable cumulative risk for AD. Based on Kaplan–Meier survival curves, AD PRS tertiles significantly increased the risk for all-cause dementia, with notable differences by sex, whereby the relationship was stronger among women (Fig. 1). This relationship was mainly detected in the contrast between T_3_ and T_1_ of AD PRS tertiles.

**Table 1:** Study sample characteristics by sex: UK biobank 2006–2021

	Overall (*N* = 40,139)	Men (*N* = 18,565)	Women (*N* = 21,574)	P_sex_
Demographic				
Baseline age, Age_base_ years	60.85 ± 0.03	61.13 ± 0.04	60.6 ± 0.04	< 0.001
Age_base_ × Age_base_	3,733.2 ± 3.3	3,768.1 ± 4.9	3,703.1 ± 4.5	< 0.001
Sex, % female	53.7%	–	–	
Race/ethnicity				
White	94.9%	94.9%	94.9%	Ref
Black	1.6%	1.4%	1.7%	0.087
South Asian	1.5%	1.8%	1.3%	0.001
Other	2.0%	1.9%	2.2%	0.041
Minority racial groups, %	5.1%	5.1%	5.1%	0.70
Genetic principal components, GPC1-20				
GPC1	-1.902 ± 0.269	-2.563 ± 0.378	-1.333 ± 0.380	0.022
GPC2	+ 1.401 ± 0.121	+ 1.386 ± 0.170	+ 1.414 ± 0.172	0.91
GPC3	-0.172 ± 0.065	0.176 ± 0.095	-0.472 ± 0.090	< 0.001
GPC4	0.113 ± 0.052	0.066 ± 0.079	+ 0.153 ± 0.070	0.41
GPC5	-0.055 ± 0.038	-0.053 ± 0.056	-0.057 ± 0.052	0.96
GPC6	-0.134 ± 0.021	-0.215 ± 0.025	-0.065 ± 0.032	< 0.001
GPC7	+ 0.047 ± 0.025	+ 0.200 ± 0.038	-0.085 ± 0.033	< 0.001
GPC8	-0.083 ± 0.023	-0.104 ± 0.031	-0.065 ± 0.032	0.40
GPC9	+ 0.098 ± 0.022	+ 0.087 ± 0.032	+ 0.011 ± 0.030	0.63
GPC10	+ 0.018 ± 0.020	+ 0.033 ± 0.032	+ 0.005 ± 0.026	0.50
GPC11	+ 0.116 ± 0.020	+ 0.153 ± 0.032	+ 0.084 ± 0.026	0.092
GPC12	+ 0.024 ± 0.019	+ 0.060 ± 0.030	-0.007 ± 0.023	0.073
GPC13	+ 0.031 ± 0.015	+ 0.024 ± 0.018	+ 0.037 ± 0.023	0.67
GPC14	+ 0.024 ± 0.017	+ 0.025 ± 0.025	+ 0.022 ± 0.023	0.93
GPC15	+ 0.019 ± 0.016	+ 0.068 ± 0.024	-0.023 ± 0.023	0.006
GPC16	+ 0.019 ± 0.016	-0.033 ± 0.024	+ 0.063 ± 0.022	0.003
GPC17	+ 0.024 ± 0.013	+ 0.000 ± 0.019	+ 0.044 ± 0.018	0.10
GPC18	+ 0.037 ± 0.014	+ 0.049 ± 0.211	+ 0.027 ± 0.020	0.45
GPC19	-0.010 ± 0.014	-0.004 ± 0.021	-0.014 ± 0.019	0.71
GPC20	+ 0.012 ± 0.014	+ 0.024 ± 0.021	+ 0.001 ± 0.019	0.42
AD PRS	+ 0.066 ± 0.005	+ 0.072 ± 0.007	+ 0.058 ± 0.007	0.18
AD PRS Tertile, %				
T1	33.3	33.3	33.4	Ref
T2	33.3	33.2	33.4	0.88
T3	33.3	33.4	33.2	0.76
Cumulative incidence, %				
All-cause dementia	2.91%	3.32%	2.55%	< 0.001
AD dementia	1.34%	1.34%	1.34	0.98

*AD* Alzheimer’s Disease; *GPC*: Genetic Principal Component; *PRS* Polygenic Risk Score; *SE* Standard Error; *UK* United Kingdom, No multiple imputation was carried out in this analysis. *P*-value is associated with the parameter for sex in bivariate linear and multinomial logistic regression analyses, with the main outcome being a continuous or categorical characteristic, respectively. (Ref) is the referent category in the multinomial logistic regression model. Values are means ± SE or percentages

Table 2 displays the estimated relationship of AD PRS score and tertile with incident dementia, using a series of Cox proportional hazards models adjusted for age, age-squared, sex and the first 20 genetic principal components. Overall, AD PRS score was associated with 79% higher risk for all-cause dementia per 1 SD increase in genetic risk in the largest UK Biobank sample (HR = 1.79, 95% CI: 1.70–1.87, *P* < 0.001). This effect was markedly stronger among women (HR = 1.96, 95% CI: 1.83–2.10, *P* < 0.001) versus men (HR = 1.64, 95% CI: 1.54–1.75, *P* < 0.001). In its tertile form, AD PRS showed a dose–response relationship with all-cause dementia in the overall sample, and among men and women, separately, with a three-fold increased risk among men and five-fold increased risk in women when comparing T_3_ to T_1_ and ~ 50% increased risk when comparing T_2_ to T_1_ in both sexes. AD PRS tertile interacted significantly with sex in its association with all-cause dementia risk (AD PRS _tert_ × sex, *P* < 0.001 in the unstratified model).

**Table 2:** Alzheimer’s Disease polygenic risk score (AD PRS) and incident dementia, overall and stratified by sex: UK biobank 2006–2021a

	AD PRS score (*N* = 40,139)	AD PRS tertile HR with 95% CI				
	AD PRS HR with 95% CI	P_AD PRS_	T_1_	T_2_	T_3_	P _AD PRStert×sex_
Overall, *N* = 40,139	**1.79** **(1.70;1.87)**	** < 0.001**	Ref	**1.53** (1.28; 1.85)***	**3.88** (3.29; 4.57)***	** < 0.001**
Men, *N* = 18,565	**1.64** **(1.54;1.75)**	**< 0.001**	Ref	**1.54** (1.22;1.96)***	**3.12** (2.51;3.87)***	n/a
Women, *N* = 21,574	**1.96** **(1.83; 2.10)**	** < 0.001**	Ref	**1.52** (1.13; 2.05)**	**5.02** (3.90; 6.46)***	n/a

*AD* Alzheimer’s Disease, *GPC* Genetic Principal Component, *HR* Hazard Ratio, *n/a* not applicable; *PRS* Polygenic Risk Score; *Ref* Referent category, UK United Kingdom

^a^All Cox proportional hazards models were adjusted for baseline age, sex, age-squared and the first 20 genetic principal components (GPC 1–20). Interaction between AD PRS tertiles and sex was tested, by including a 2-way interaction term in the main unstratified model. Values are hazard ratios with 95% CI. 1 SD of AD PRS is equivalent to a 1 unit increase in AD PRS in the largest available UK Biobank sample. **P<0.010; ***P<0.001 for null hypothesis that Ln(HR)=0

**Fig. 2:**
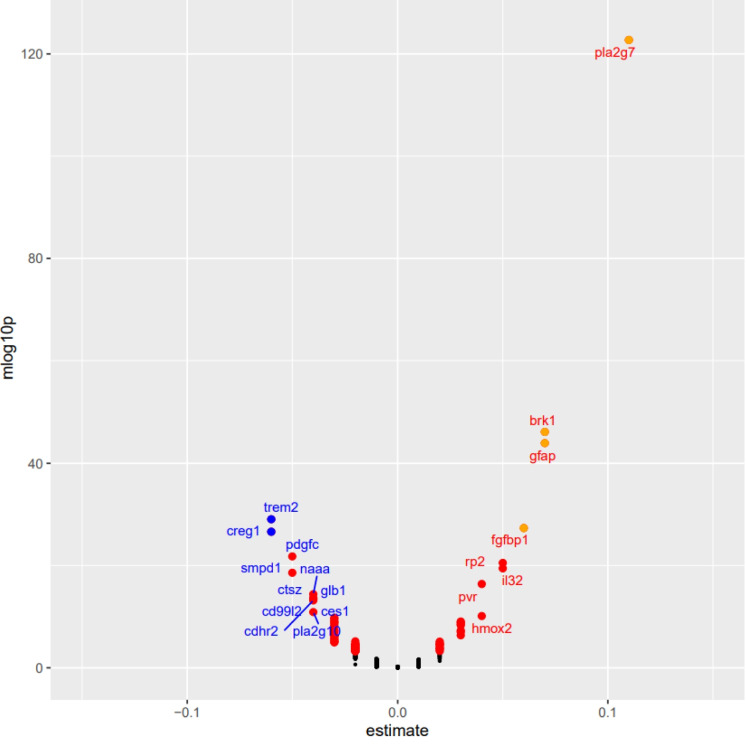
Volcano plot of plasma proteomic biomarkers in relation to AD PRS: UK biobank 2006–2010. *Abbreviations*: See list of abbreviations for protein abbreviations and https://www.ncbi.nlm.nih.gov/gene/; AD = Alzheimer’s Disease; PRS = Polygenic Risk Score. *Note*: Based on a series of multiple linear regression models, with main predictor being AD PRS and the outcome being each of 1,463 plasma proteomic biomarkers (Log2 transformed, z-scored). The y-axis is the predictor’s associated *p*-value on a -Log10 scale and the X-axis is the β coefficient (effect of AD PRS exposure on standardized z-scores of plasma proteomic markers) from the multiple linear regression models. An estimate with a Bonferroni corrected *p*-value < 0.05 are marked by a different color and the plasma proteomic marker abbreviation is added for relatively stronger effect size of > 0.050 in absolute value (See UKB showcase URL: https://biobank.ndph.ox.ac.uk/showcase/). Selected proteins (*k* = 86) for further mediation analysis have a corrected *p*-value < 0.05. Details are provided on github: https://github.com/baydounm/UKB-paper12-supplementarydata

All 1,463 least square regression analyses were performed to determine the strongest relationships between AD PRS score, the main exposure, and the plasma proteome. Following Bonferroni adjustment, AD PRS was associated significantly with 86 plasma proteins (Fig. 2). Figure 2 shows that PLA2G7 had the largest positive effect size, followed by BRK1 and the glial fibrillary acidic protein (GFAP), while TREM2 had the strongest inverse association with AD PRS, followed closely by CREG1 and PDGFC.

**Fig. 3:**
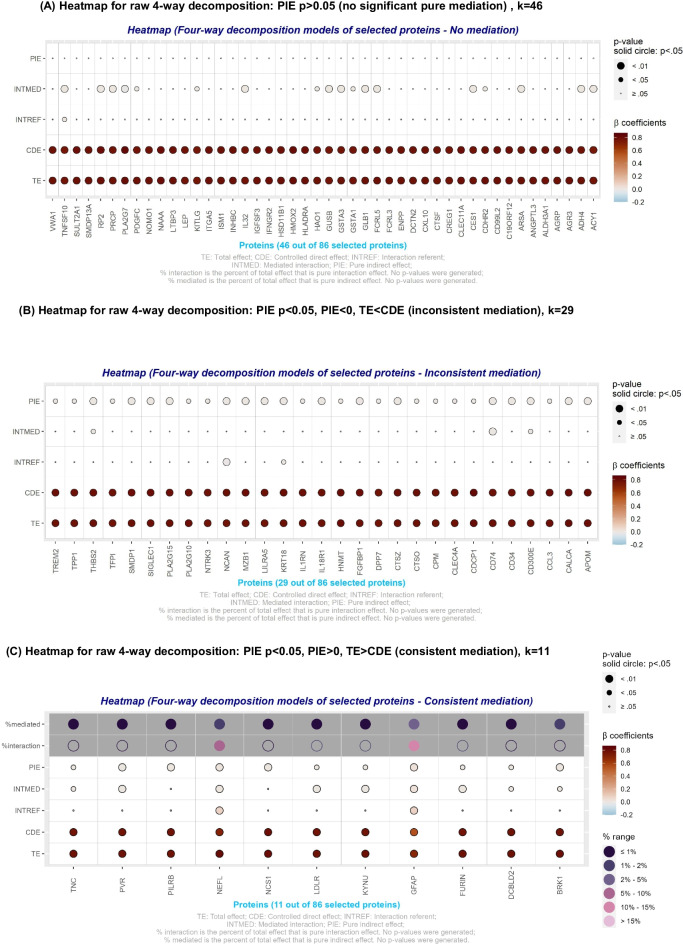
Four-way decomposition of the association between Alzheimer’s Disease polygenic risk score and incidence of all-cause dementia by the selected plasma proteomic biomarkers (*k* = 86): UK biobank 2006–2021. (**A**) Heatmap for 4-way decomposition: PIE *p* > 0.05 (no significant pure mediation), *k* = 45. (**B**) Heatmap for 4-way decomposition: PIE *p* < 0.05, PIE < 0, TE < CDE (inconsistent mediation), *k* = 30. (**C**) Heatmap for 4-way decomposition: PIE *p* < 0.05, PIE > 0, TE > CDE (consistent mediation), *k* = 11. Note: Effects could range from -0.20 to 0.80 . P values are ranked as > or =0.05, <0.05 or <0.010. *P < 0.05; **P < 0.010;***P < *0.001. Abbreviations*: ereri_cde = excess relative risk due to neither mediation nor interaction or controlled direct effect; ereri_intmed = excess relative risk due to mediated interaction or mediated interaction; ereri_intref = excess relative risk due to interaction only or reference interaction; ereri_pie = excess relative risk due to mediation only or pure indirect effect; tereri = Total excess relative risk; UK = United Kingdom. See supplementary Table 5 (Appendix II) for protein abbreviations. Other Protein abbreviations are found at https://www.ncbi.nlm.nih.gov/gene/

Four-way decomposition models applied to selected proteins with substantial correlation to the primary exposure (*k* = 86) are presented in Table 3, Fig. 3, and Appendix V (supplementary datasheet 1). The results were divided into three groups, as Fig. 3 illustrates: focusing on the significance of PIE and its direction in relation to TE, there was (A) no mediation (*P* > 0.05); (B) inconsistent mediation (*P* < 0.05 for PIE with |CDE| >|TE| and PIE sign that differs from TE); and (C) consistent mediation (*P* < 0.05 for PIE with |CDE| <|TE| and PIE sign that is the same as TE). Table 3 presents findings for consistent mediation, while Appendix V (supplementary datasheet 1) has the full set of findings. The results showed that just 11 of the selected proteins were consistent mediators (i.e., group C), whereas most proteins (*k* = 46: i.e., group A) were not significant mediators. A sizable number of the proteins (*k* = 29: i.e., group B) were inconsistent mediators. In both groups B and C, percentages mediated were small (< 5% were PIE) for all proteins, with most having either < 1% or between 1 and 5%. Nevertheless, two notable findings with respect to combined mediation and moderation were observed, both in group C. First, GFAP, one of the plasma proteins most strongly related to AD PRS, was a statistically significant mediator and moderator in the relationship between AD PRS and all-dementia, with pure interaction (INTREF) accounting for 10–15% of the TE, in addition to PIE accounting for 2–5% of the TE. Similarly, neurofilament light chain (NfL; gene name *NEFL*), while mediating a small percentage of TE, interacted significantly with AD PRS in relation to all-cause dementia, with INTREF of NfL, accounting for 5–10% of the AD PRS-dementia TE.

**Table 3:** Four-way decomposition of the association between AD PRS z-score and all-cause dementia through selected plasma proteomic biomarkers (*k* = 11; *N*_max_ = 40,139, with consistent mediation): UK biobank 2006–2021

FOURWAYDECOMP	Beta	SE	z	*P*	LCL	UCL	Protein
te	**0.7928**	**0.0439**	**18.06**	** < 0.001**	**0.7068**	**0.8788**	BRK1
cde	**0.7735**	**0.0446**	**17.33**	** < 0.001**	**0.6860**	**0.8610**	BRK1
intref	0.0044	0.0037	1.2	0.229	-0.0028	0.0116	BRK1
intmed	**0.0065**	**0.0026**	**2.5**	**0.012**	**0.0014**	**0.0117**	BRK1
pie	**0.0083**	**0.0027**	**3.07**	**0.002**	**0.0030**	**0.0137**	BRK1
p_cde	**0.9757**	**0.0092**	**105.98**	** < 0.001**	**0.9576**	**0.9937**	BRK1
p_intref	0.0055	0.0047	1.19	0.233	-0.0036	0.0147	BRK1
p_intmed	**0.0082**	**0.0033**	**2.47**	**0.014**	**0.0017**	**0.0148**	BRK1
p_pie	**0.0105**	**0.0034**	**3.07**	**0.002**	**0.0038**	**0.0172**	BRK1
te	**0.6861**	**0.0443**	**15.51**	** < 0.001**	**0.5994**	**0.7728**	GFAP
cde	**0.5509**	**0.0442**	**12.47**	** < 0.001**	**0.4643**	**0.6374**	GFAP
intref	**0.0824**	**0.0096**	**8.62**	** < 0.001**	**0.0637**	**0.1012**	GFAP
intmed	**0.0260**	**0.0026**	**10.02**	** < 0.001**	**0.0209**	**0.0310**	GFAP
pie	**0.0268**	**0.0028**	**9.5**	** < 0.001**	**0.0213**	**0.0324**	GFAP
p_cde	**0.8029**	**0.0208**	**38.65**	** < 0.001**	**0.7622**	**0.8436**	GFAP
p_intref	**0.1202**	**0.0156**	**7.72**	** < 0.001**	**0.0896**	0.1507	GFAP
p_intmed	**0.0378**	**0.0038**	**10.07**	** < 0.001**	**0.0305**	0.0452	GFAP
p_pie	**0.0391**	**0.0044**	**8.84**	** < 0.001**	**0.0304**	0.0477	GFAP
te	**0.7800**	**0.0423**	**18.42**	** < 0.001**	**0.6970**	**0.8630**	PVR
cde	**0.7638**	**0.0424**	**18.01**	** < 0.001**	**0.6807**	**0.8470**	PVR
intref	***0.0066***	***0.0035***	***1.9***	***0.058***	***-0.0002***	***0.0135***	PVR
intmed	**0.0047**	**0.0014**	**3.36**	**0.001**	**0.0020**	**0.0074**	PVR
pie	**0.0048**	**0.0017**	**2.91**	**0.004**	**0.0016**	**0.0081**	PVR
p_cde	**0.9793**	**0.0071**	**138.54**	** < 0.001**	**0.9654**	**0.9931**	PVR
p_intref	***0.0085***	***0.0045***	***1.89***	***0.059***	***-0.0003***	***0.0173***	PVR
p_intmed	**0.0060**	**0.0018**	**3.36**	**0.001**	**0.0025**	**0.0095**	PVR
p_pie	**0.0062**	**0.0021**	**2.89**	**0.004**	**0.0020**	**0.0104**	PVR
te	**0.8057**	**0.0462**	**17.46**	** < 0.001**	**0.7153**	**0.8962**	NEFL
cde	**0.7006**	**0.0460**	**15.24**	** < 0.001**	**0.6105**	**0.7906**	NEFL
intref	**0.0788**	**0.0125**	**6.32**	** < 0.001**	**0.0544**	**0.1033**	NEFL
intmed	**0.0120**	**0.0022**	**5.55**	** < 0.001**	**0.0077**	**0.0162**	NEFL
pie	**0.0143**	**0.0024**	**6.06**	** < 0.001**	**0.0097**	**0.0190**	NEFL
p_cde	**0.8695**	**0.0180**	**48.27**	** < 0.001**	**0.8342**	**0.9048**	NEFL
p_intref	**0.0979**	**0.0158**	**6.18**	** < 0.001**	**0.0668**	**0.1289**	NEFL
p_intmed	**0.0149**	**0.0026**	**5.77**	** < 0.001**	**0.0098**	**0.0199**	NEFL
p_pie	**0.0178**	**0.0030**	**6.00**	** < 0.001**	**0.0120**	**0.0236**	NEFL
te	**0.7811**	**0.0423**	**18.48**	** < 0.001**	**0.6982**	**0.8639**	FURIN
cde	**0.7666**	**0.0425**	**18.05**	** < 0.001**	**0.6834**	**0.8498**	FURIN
intref	***0.0087***	***0.0047***	***1.82***	***0.068***	***-0.0006***	***0.0180***	FURIN
intmed	**0.0036**	**0.0013**	**2.88**	**0.004**	**0.0012**	**0.0061**	FURIN
pie	**0.0022**	**0.0011**	**1.98**	**0.048**	**0.0000**	**0.0044**	FURIN
p_cde	**0.9814**	**0.0081**	**121.36**	** < 0.001**	**0.9656**	**0.9973**	FURIN
p_intref	***0.0111***	***0.0061***	***1.82***	***0.069***	***-0.0009***	***0.0230***	FURIN
p_intmed	**0.0046**	**0.0016**	**2.88**	**0.004**	**0.0015**	**0.0078**	FURIN
p_pie	**0.0028**	**0.0014**	**1.97**	**0.049**	**0.0000**	**0.0057**	FURIN
te	**0.7769**	**0.0424**	**18.33**	** < 0.001**	**0.6938**	**0.8600**	LDLR
cde	**0.7624**	**0.0423**	**18.01**	** < 0.001**	**0.6795**	**0.8454**	LDLR
intref	***0.0087***	***0.0049***	***1.78***	***0.075***	***-0.0009***	***0.0183***	LDLR
intmed	**0.0032**	**0.0012**	**2.75**	**0.006**	**0.0009**	**0.0055**	LDLR
pie	**0.0025**	**0.0011**	**2.4**	**0.016**	**0.0005**	**0.0046**	LDLR
p_cde	**0.9814**	**0.0081**	**121.06**	** < 0.001**	**0.9655**	**0.9972**	LDLR
p_intref	***0.0112***	***0.0063***	***1.79***	***0.074***	***-0.0011***	***0.0235***	LDLR
p_intmed	**0.0042**	**0.0015**	**2.77**	**0.006**	**0.0012**	**0.0071**	LDLR
p_pie	**0.0033**	**0.0014**	**2.39**	**0.017**	**0.0006**	0.0059	LDLR
te	**0.7975**	**0.0433**	**18.42**	** < 0.001**	**0.7127**	**0.8823**	NCS1
cde	**0.7922**	**0.0438**	**18.07**	** < 0.001**	**0.7063**	**0.8781**	NCS1
intref	0.0002	0.0036	0.07	0.947	-0.0069	0.0074	NCS1
intmed	0.0013	0.0009	1.47	0.141	-0.0004	0.0031	NCS1
pie	**0.0037**	**0.0011**	**3.26**	**0.001**	**0.0015**	**0.0060**	NCS1
p_cde	**0.9933**	**0.0058**	**171.63**	** < 0.001**	**0.9820**	**1.0047**	NCS1
p_intref	0.0003	0.0046	0.07	0.947	-0.0086	0.0092	NCS1
p_intmed	0.0017	0.0011	1.47	0.142	-0.0006	0.0039	NCS1
p_pie	**0.0047**	**0.0014**	**3.26**	**0.001**	**0.0019**	**0.0075**	NCS1
te	**0.7772**	**0.0424**	**18.33**	** < 0.001**	**0.6941**	**0.8603**	TNC
cde	**0.7685**	**0.0426**	**18.04**	** < 0.001**	**0.6850**	**0.8520**	TNC
intref	0.0048	0.0035	1.36	0.175	-0.0021	0.0117	TNC
intmed	**0.0020**	**0.0009**	**2.2**	**0.028**	**0.0002**	**0.0037**	TNC
pie	**0.0019**	**0.0009**	**2.19**	**0.029**	**0.0002**	**0.0037**	TNC
p_cde	**0.9888**	**0.0060**	**166.12**	** < 0.001**	**0.9772**	**1.0005**	TNC
p_intref	0.0061	0.0045	1.35	0.176	-0.0028	0.0151	TNC
p_intmed	**0.0025**	**0.0012**	**2.2**	**0.028**	**0.0003**	**0.0048**	TNC
p_pie	**0.0025**	**0.0011**	**2.18**	**0.029**	**0.0003**	**0.0047**	TNC
te	**0.7854**	**0.0424**	**18.51**	** < 0.001**	**0.7022**	**0.8685**	KYNU
cde	**0.7713**	**0.0423**	**18.22**	** < 0.001**	**0.6883**	**0.8542**	KYNU
intref	***0.0092***	***0.0050***	***1.84***	***0.066***	***-0.0006***	***0.0190***	KYNU
intmed	**0.0028**	**0.0011**	**2.65**	**0.008**	**0.0007**	**0.0049**	KYNU
pie	**0.0021**	**0.0009**	**2.2**	**0.028**	**0.0002**	**0.0039**	KYNU
p_cde	**0.9821**	**0.0079**	**124.35**	** < 0.001**	**0.9666**	**0.9975**	KYNU
p_intref	***0.0117***	***0.0063***	***1.85***	***0.065***	***-0.0007***	***0.0242***	KYNU
p_intmed	**0.0036**	**0.0013**	**2.68**	**0.007**	**0.0010**	**0.0062**	KYNU
p_pie	**0.0026**	**0.0012**	**2.19**	**0.029**	**0.0003**	**0.0050**	KYNU
te	**0.7788**	**0.0427**	**18.26**	** < 0.001**	**0.6952**	**0.8624**	PILRB
cde	**0.7748**	**0.0427**	**18.14**	** < 0.001**	**0.6911**	**0.8585**	PILRB
intref	0.0000	0.0036	0.01	0.992	-0.0070	0.0071	PILRB
intmed	0.0010	0.0008	1.2	0.228	-0.0006	0.0026	PILRB
pie	**0.0030**	**0.0011**	**2.8**	**0.005**	**0.0009**	**0.0050**	PILRB
p_cde	**0.9949**	**0.0057**	**175.35**	** < 0.001**	**0.9838**	**1.0060**	PILRB
p_intref	0.0000	0.0046	0.01	0.992	-0.0090	0.0091	PILRB
p_intmed	0.0013	0.0011	1.21	0.227	-0.0008	0.0034	PILRB
p_pie	**0.0038**	**0.0014**	**2.79**	**0.005**	**0.0011**	**0.0065**	PILRB
te	**0.7858**	**0.0434**	**18.09**	** < 0.001**	**0.7007**	**0.8710**	DCBLD2
cde	**0.7765**	**0.0440**	**17.66**	** < 0.001**	**0.6903**	**0.8627**	DCBLD2
intref	0.0051	0.0040	1.27	0.204	-0.0028	0.0129	DCBLD2
intmed	**0.0019**	**0.0009**	**2.18**	**0.029**	**0.0002**	**0.0036**	DCBLD2
pie	**0.0023**	**0.0009**	**2.54**	**0.011**	**0.0005**	**0.0042**	DCBLD2
p_cde	**0.9882**	**0.0064**	**153.61**	** < 0.001**	**0.9755**	**1.0008**	DCBLD2
p_intref	0.0065	0.0051	1.26	0.208	-0.0036	0.0165	DCBLD2
p_intmed	**0.0024**	**0.0011**	**2.16**	**0.031**	**0.0002**	**0.0046**	DCBLD2
p_pie	**0.0030**	**0.0012**	**2.55**	**0.011**	**0.0007**	**0.0053**	DCBLD2

*AD* Alzheimer’s Disease; ereri_cde = excess relative risk due to neither mediation nor interaction or controlled direct effect; ereri_intmed = excess relative risk due to mediated interaction or mediated interaction; ereri_intref = excess relative risk due to interaction only or reference interaction; ereri_pie = excess relative risk due to mediation only or pure indirect effect; p_cde = proportion of total effect that is controlled direct effect; p_intmed = proportion of total effect that is mediated interaction; p_intref = proportion of total effect that is reference interaction; p_pie = proportion of total effect that is pure indirect effect; tereri = Total excess relative risk; PRS = Polygenic Risk Score; UK = United Kingdom. Names of the genes/proteins can be found at: https://www.ncbi.nlm.nih.gov/gene. Tereri and ereri_cde are interpreted as Log_e_(hazard ratios). AD PRS is originally normalized as a Z-score, and therefore a unit increase is equivalent to 1 SD increase in AD polygenic risk. Models were adjusted for age, age-squared, sex and the first 20 genetic principal components (GPC 1–20)

In secondary analyses with 2022 AD PRS[26], those results were largely replicated with a much greater % pure interaction (34% for GFAP and 7% for NfL), but with comparable % pure mediation (2–5% for each of these plasma proteins) for AD PRS-dementia TE, particularly for NfL and GFAP proteins [Appendix V (supplementary datasheet 1) and full Output in https://github.com/baydounm/UKB-paper12-supplementarydata]. It is worth noting that the 2022 AD PRS[26] correlated weakly to moderately with 2019 IGAP[24] AD PRS (Pearson’s *r* = 0.29, *p* < 0.001) and that unlike the main AD PRS (HR = 1.8 per SD), the newer version was associated with approximately 24% increase in dementia risk overall per SD. Other findings are presented in their respective OSMs, including principal component analyses (OSM 4 within Appendix II), Olink Insight and STRING analyses (OSM 7 within Appendix II and supplementary datasheet 2 or Appendix IX). Other secondary analyses are described in OSM 8 within Appendix II and supplementary datasheet 3 (or Appendix XII) with full Output provided in https://github.com/baydounm/UKB-paper12-supplementarydata. Most notably, we found a significantly stronger association between AD PRS and all-cause dementia, with the main AD PRS incorporating APOE SNPs. We also found that mediator 2 (which loaded highly on GFAP and NEFL) was the main PCA component that explained a large proportion of the TE, mainly by interaction reference but also by pure indirect effect and mediated interaction for both all-cause and AD dementia outcomes, and for both the main (IGAP 2019) and new AD PRS (2022). Findings are summarized in Appendix XI (Figure S5). 

## Discussion

This large (*n* = 40,139) retrospective cohort study of older individuals found that AD PRS tertiles significantly increased all-cause dementia risk, with stronger relationships among women. The study found that AD PRS score was associated with 79% higher risk for all-cause dementia. There were 86 plasma proteins, including PLA2G7, BRK1, GFAP, and TREM2, that were significantly predicted by AD PRS. Both the glial protein GFAP and the neuronal protein NfL significantly interacted synergistically with AD PRS to increase all-dementia risk (> 10% of TE is pure interaction), while GFAP was also an important consistent mediator in the AD PRS-dementia relationship. Thus, two potential blood-derived markers of neurological disorders and diseases are important mediators and moderators of the relationship between AD PRS and all-cause dementia.

Over time, numerous versions of AD PRS have been derived from extensive genome-wide association studies (GWAS). These scores have recently been confirmed through assessment against other indicators of brain degeneration, such as volume shrinkage of the hippocampus and precuneus, as well as cognitive impairment associated with aging [36–39]. In a prior investigation, older adults at risk for dementia (2006–2010) from the Health and Retirement Study (*n* = 7,690, 50 +) was examined [40]. The study revealed that the measurement of AD PRS using 10 AD polymorphisms was more significantly linked to dementia onset in NHW individuals compared to NHB individuals [40]. In a more recent study using HRS data, the follow-up period was increased to 2010–2018 and employed different definitions for AD and all-cause dementia [14]. Findings from this study revealed a correlation between AD PRS (IGAP-2019) and ADRD, but this association was only observed among non-Hispanic white individuals [14]. Furthermore, the study detected significant variations between sexes, with women showing a more pronounced impact of AD PRS on AD incidence compared to men (*p* < 0.05 for AD PRS × sex interaction), even after accounting for race and other factors [14]. A recent cohort study discovered the influence of sex-dependent autosomal effects on the clinical course of AD [13]. Additionally, earlier research demonstrated that the relationship between APOE ε4 allele and AD incidence is more pronounced in women [12]. We opted for an AD PRS incorporating the APOE ε4 allele. A recent study has demonstrated that this PRS carries further benefits when compared with the APOE ε4 allele in predicting AD and distinguishing it from other types of dementia [39]. The greater genetic risk for all-cause dementia observed among women, although documented in other studies, has not been examined in depth so as to explain the biological mechanisms behind it.

Two important moderators and mediators of the association between AD PRS and all-cause dementia were detected in our study and those were neurofilament light chain (NfL) and glial acidic fibrillary protein (GFAP). Blood NfL is a marker of neuroaxonal damage whereas blood GFAP may reflect astrocyte activation or damage in the central nervous system. Associations of NfL and GFAP with dementia and other related traits has been previously documented. Most notably, as a predictive and susceptibility biomarker for AD-related neurodegeneration in clinical and research settings, blood NfL has demonstrated promise as a blood-based biomarker for AD[41]. When neurons suffer axonal injury, cytoskeletal proteins like neurofilaments may be released into the extracellular space, CSF, and eventually the blood at a reduced concentration [42]. Researchers have discovered predictive plasma biomarkers for AD and evaluated how well these biomarkers predict the disease status [43]. Most notably, epsilon4, Abeta40 or Abeta42, GFAP, and NfL were the most important contributors to the prediction accuracy of AD clinical diagnosis, which was reported to attain AUC = 0.81[43]. GFAP levels were higher in the Abeta-positive group, in AD, and in moderate cognitive impairment than in healthy controls, according to a systematic review and meta-analysis[44]. Clinical measurement of blood GFAP can accelerate AD diagnosis and enhance prognosis[44]. Importantly both NfL and GFAP were shown to be important independent predictors for dementia and sub-types in a very recent study using comparable data form the UK Biobank cohort [21].

Several studies examined NfL and GFAP in relation to AD PRS or their interaction with AD PRS in relation to dementia risk [45–47]. In a nested case–control study, APOE4 status and polygenic risk were significantly associated to levels of blood GFAP [47]. In another large study conducted in China, AD PRS was correlated with plasma Abeta42, Abeta42/Abeta40 ratio, T-tau, and NfL levels [45]. Interaction between AD PRS and NfL was detected in a third study, whereby AD PRS combined with baseline plasma NfL predicted onset of AD (*p* < 0.01), and NfL correlated with AD PRS within AD cases [46]. Our present study showed that both NfL and GFAP interacted synergistically with AD PRS to determine the risk for all-cause dementia. To our knowledge, no other study has detected such consistent interactions between AD PRS and those two plasma proteins in relation to all-cause dementia.

Among all plasma proteins tested against AD PRS, two showed strongest positive and inverse relationships, namely phospholipase A2 group 7, also known as platelet activating factor acetyl hydrolase, (PLA2G7) and triggering receptor expressed on myeloid cells 2 (TREM2), respectively. While PLA2G7 was not a mediator, it was among several proteins exhibiting a weak mediated interaction with AD PRS. In contrast, TREM2 showed a weak inconsistent mediation based mainly on a statistically significant PIE. Nevertheless, both of these proteins did not lead to a significant change in the TE. PLA2G7 is a lipoprotein-associated phospholipase A2 that was recently implicated in regulating metabolism, inflammation and lifespan in response to caloric restriction in mice and humans [48]. TREM2 is a triggering receptor that is expressed in myeloid cells, including microglia in the CNS, with roles in regulating phagocytosis, anti-inflammatory responses and proliferation and survival [49]. While less evidence is available for a causal relationship *PLA2G7* and AD[50], *TREM2* gene has been linked to AD in numerous recent studies, ranking second according to a recent review and meta-analysis in importance after amyloids/tau [51]. Evidence in the literature regarding the association of the 11 strongest consistent mediators that were detected in this study with both AD PRS (or the APOE genotype) and all-cause dementia as well as neurodegeneration is outlined in Appendix II, OSM 6 within Appendix II (Supplementary Table 5).

Our present study includes some noteworthy strengths as well as limitations. This is a wide-scale proteomic investigation examining the relationship between AD PRS and dementia in a large cohort. Second, outcome variables were created using diagnostic dates obtained through record linkage. Third, a wide range of participants are included in the UK Biobank, which makes it possible to evaluate exposure-outcome connections objectively through confounder correction. Selection bias resulting from missing data is a possible research limitation. Moreover, the precise onset of dementia is uncertain, as is the case with most investigations. Due to the observational nature of this study, residual confounding is possible even if many confounders were taken into consideration. Moreover, reverse causality is still a plausible hypothesis even after cases of prevalent dementia were removed. Four-way decomposition models implementation makes strong assumptions, including the absence of unmeasured confounding (Appendix IV) [52]. Furthermore, *med4way* command does not have means to reduce the risk of exposure-induced mediator-outcome confounding, which cannot be completely ruled out [29]. Healthy volunteer bias provides another potential limitation to using UK Biobank data [53, 54]. The UK Biobank may not be entirely representative of the country's population, and our studies lacked the power to stratify according to specific racial or ethnic groupings. Nevertheless, we have included genetic principal components in our analyses as an attempt to control models for ethnicity and population stratification in a more granular manner.

In summary, there was a significant interplay of NfL and GFAP with AD PRS in relation to dementia incidence among UK older adults, mainly as pure interaction. The synergism between AD PRS and GFAP (and NfL) in relation to dementia risk suggests that both genetic predisposition and specific biomarkers increase the risk of dementia. Combining genetic risk and biomarker expression could improve early detection and intervention strategies. The molecular mechanisms behind interaction between AD PRS and GFAP particularly could offer insights into dementia pathophysiology and therapeutic targets. This finding, pending further replication, could also inform personalized dementia prevention and management, particularly for individuals with high AD PRS and elevated GFAP levels.

## Electronic supplementary material 

Below is the link to the electronic supplementary material.

Supplementary file1 Appendix I – Author contributions (PDF 663. KB)

Supplementary file2 Appendix II – Online Supplemental Materials (PDF 331 KB)

Supplementary file3 Appendix III – Supplementary Figure S1 (PDF 57.7 KB)

Supplementary file4 Appendix IV– Four-way decomposition models appendix (PDF 176 KB )

Supplementary file5 Appendix V – Supplementary datasheet 1 (XLSX 76.7 KB )

Supplementary file6 Appendix VI – Supplementary Figure S2 (PDF 97.0 KB)

Supplementary file7 Appendix VII – Supplementary Figure S3 (PDF 170 KB)

Supplementary file8 Appendix VIII– Supplementary Figure S4 (PDF 0.98 MB)

Supplementary file9 Appendix IX – Supplementary datasheet 2 (XLSX 29.3 KB)

Supplementary file10 Appendix X – Key mediators (TXT 1.72 KB)

Supplementary file11 Appendix XI – Supplementary Figure S5 (PDF 586 KB)

Supplementary file12 Appendix XII – Supplementary datasheet 3 (XLSX 79.5 KB)

## Data Availability

These data are subject to the following licenses and restrictions: UK Biobank is a large-scale biomedical database and research resource containing in-depth genetic and health information from about 500,000 United Kingdom participants. The data are augmented regularly with additional records and are globally accessible to approved researchers undertaking vital research into the most common and life-threatening diseases. Requests to access these datasets should be directed to https://www.ukbiobank.ac.uk/.

## Abbreviations

AD: Alzheimer’s disease
Aβ: Amyloid beta
CI: Confidence interval
CNS: Central nervous system
ereri_cde: Excess relative risk due to neither mediation nor interaction or controlled direct effect
ereri_intmed: Excess relative risk due to mediated interaction or mediated interaction
ereri_intref: Excess relative risk due to interaction only or reference interaction
ereri_pie: Excess relative risk due to mediation only or pure indirect effect
GFAP: Glial fibrillary acidic protein
GPC: Genetic principal component
HR: Hazard ratio
ICD: 10 International Classification of Diseases, Tenth Revision
MRI: Magnetic resonance imaging
NEFL: NfL gene name
NfL: Neurofilament light chain
OLS: Ordinary lease squares
PCA: Principal component analysis
pct_cde: Percent of total effect that is controlled direct effect
pct_intmed: Percent of total effect that is mediated interaction
pct_intref: Percent of total effect that is reference interaction
pct_pie: Percent of total effect that is pure indirect effect
PC: Principal component
PH: Proportional hazards
PIE: Pure indirect effects
PRS: Polygenic risk score
Ref: Referent category
SD: Standard deviation
TDI: Townsend Deprivation Index
tereri: Total excess relative risk
UK: United Kingdom
UKB-PPP: UK Biobank Pharma Proteomics Project

## Code availability

All codes and related results datasets will be made available on github at https://github.com/baydounm/UKB-paper12-supplementarydata.
