# Supplementary material for: Alzheimer’s Disease polygenic risk, the plasma proteome, and dementia incidence among UK older adults
Source: GeroScience. 2024 Nov 26;47(2):2507–23. doi: 10.1007/s11357-024-01413-8 (PMC11978584; doi:10.1007/s11357-024-01413-8)
Supplement: Supplementary file 1 — Supplementary file1 Appendix I – Author contributions (PDF 663. KB) [file 11357_2024_1413_MOESM1_ESM.pdf]

**Appendix I – Authors contributions**

| **Name** | **Location** | **Contributions** |
|---|---|---|
| May A. Beydoun, PhD | *Laboratory of Epidemiology and Population Sciences, National Institute on Aging, NIA/NIH/IRP, Baltimore, MD, USA 21224* | Study concept, data acquisition, plan of analysis, data management and statistical analysis, literature search and review, write-up of parts of the manuscript, revision of the manuscript. |
| Hind A. Beydoun, PhD | *Laboratory of Epidemiology and Population Sciences, National Institute on Aging, NIA/NIH/IRP, Baltimore, MD, USA 21224* | Study concept, plan of analysis, assistance with data management and statistical analysis, literature search and review, write-up of parts of the manuscript, revision of the manuscript. |
| Zhiguang Li | *Laboratory of Epidemiology and Population Sciences, National Institute on Aging, NIA/NIH/IRP, Baltimore, MD, USA 21224* | Study concept, plan of analysis, assistance with data management and statistical analysis, literature search and review, write-up of parts of the manuscript, revision of the manuscript. |

| | | |
|---|---|---|
| Yi-Han Hu | *Laboratory of Epidemiology and Population Sciences, National Institute on Aging, NIA/NIH/IRP, Baltimore, MD, USA 21224* | Study concept, plan of analysis, literature search and review, data visualization, review of the manuscript. |
| Nicole Noren Hooten, PhD | *Laboratory of Epidemiology and Population Sciences, National Institute on Aging, NIA/NIH/IRP, Baltimore, MD, USA 21224* | Study concept, plan of analysis, literature search and review, write-up of parts of the manuscript, review of the manuscript. |
| Jun Ding | *Translational Gerontology Branch, National Institute on Aging, NIA/NIH/IRP, Baltimore, MD, USA 21224* | Study concept, plan of analysis, literature search and review, write-up of parts of the manuscript, review of the manuscript. |
| Sharmin Hossain, PhD | *Laboratory of Epidemiology and Population Sciences, National Institute on Aging, NIA/NIH/IRP, Baltimore, MD, USA 21224* | Plan of analysis, literature search and review, write-up of parts of the manuscript, revision of the manuscript. |
| Christian A. Maino Vieytes, PhD | *Laboratory of Epidemiology and Population Sciences, National Institute on Aging, NIA/NIH/IRP, Baltimore, MD, USA 21224* | Plan of analysis, assistance with data management, data visualization, write-up of parts of the manuscript, literature search and review, revision of the manuscript. |

| | | |
|---|---|---|
| Lenore J. Launer, PhD | *Laboratory of Epidemiology and Population Sciences, National Institute on Aging, NIA/NIH/IRP, Baltimore, MD, USA 21224* | Plan of analysis, literature search and review, write-up of parts of the manuscript, revision of the manuscript. |
| Michele K. Evans, PhD | *Laboratory of Epidemiology and Population Sciences, National Institute on Aging, NIA/NIH/IRP, Baltimore, MD, USA 21224* | Data acquisition, plan of analysis, write-up of parts of the manuscript, revision of the manuscript. |
| Alan B. Zonderman, PhD | *Laboratory of Epidemiology and Population Sciences, National Institute on Aging, NIA/NIH/IRP, Baltimore, MD, USA 21224* | Data acquisition, plan of analysis, write-up of parts of the manuscript, revision of the manuscript. |
