# Supplementary material for: Alzheimer’s Disease polygenic risk, the plasma proteome, and dementia incidence among UK older adults
Source: GeroScience. 2024 Nov 26;47(2):2507–23. doi: 10.1007/s11357-024-01413-8 (PMC11978584; doi:10.1007/s11357-024-01413-8)
Supplement: Supplementary file 2 — Supplementary file2 Appendix II – Online Supplemental Materials (PDF 331 KB) [file 11357_2024_1413_MOESM2_ESM.pdf]

APPENDIX II

ONLINE SUPPLEMENTARY MATERIALS

Alzheimer's Disease polygenic risk, the plasma proteome and dementia incidence among UK older adults

May A. Beydoun . al

OSM1: AD PRS, IGAP 2019 and genetic principal components

AD PRS: Details on the genomic component of UK Biobank is provided elsewhere(1). PRS scores were developed and applied to meta-analyzed (and, where possible, ancestry specific) GWAS summary statistics that were either completely extracted from external GWAS data (the Standard PRS set) or from a combination of external and internal UK Biobank data (the Enhanced PRS set) using a Bayesian approach. The Standard PRS Set (also known as the "UKB-Free" set), which includes 28 diseases and 8 quantitative traits, was generated using external GWAS data; Thompson et al. described the method in the supplementary material for the main paper in 2022 (https://www.medrxiv.org/content/10.1101/2022.06.16.22276246v1.supplementary-material?versioned=true). From the list of standard PRS, we selected AD PRS, which was originally located in the PGS catalog (https://www.pgscatalog.org). We chose a version of AD PRS for our main analysis that included APOE single nucleotide polymorphisms (SNPs), based on the IGAP-2019 GWAS results. Details are provided in: PRS_Release_README.pdf (ox.ac.uk). In this study we have focused on the Standard PRS set which was generated independently of the UK Biobank dataset and therefore using external data for the GWAS results exclusively.

IGAP-2019: An extensive three-stage study called the International Genomics of Alzheimer's Project (IGAP) is based on genome-wide association studies (GWAS) conducted on people with European ancestry(2). In the first stage, IGAP meta-analyzed GWAS datasets from four consortia: The Alzheimer Disease Genetics Consortium (ADGC); The European Alzheimer's disease Initiative (EADI); The Cohorts for Heart and Aging Research in Genomic Epidemiology Consortium (CHARGE); and The Genetic and Environmental Risk in AD Consortium Genetic and Environmental Risk in AD/Defining Genetic, Polygenic and Environmental Risk for Alzheimer's Disease Consortium (GERAD/PERADES)(2). The genotyped and imputed data on 11,480,632 single nucleotide polymorphisms (SNPs) were used in the first stage of IGAP. In stage 2, 10,483 controls and 8,362 Alzheimer's disease cases were used as the independent sample for genotyping and association testing on 11,632 SNPs.

Following a meta-analysis of variations chosen for analysis in either the stage 3A (n = 11,666) or stage 3B (n = 30,511) samples, 35,274 clinical and autopsy-documented cases of Alzheimer's disease and 59,163 controls made up the final sample(2). Details of genes and SNPs included at each stage are in the following URL: Genetic meta-analysis of diagnosed Alzheimer's disease identifies new risk loci and implicates Aβ, tau, immunity and lipid processing (nih.gov)

Genetic principal components:

Top genetic principal components that explain the most variation were estimated fastPCA38 that performs well on datasets with hundreds of thousands of samples(1). Using a set of 407,219 unrelated, high-quality samples and 147,604 high-quality markers that had been trimmed to reduce linkage disequilibrium, top 40 main components were calculated(1), of which the first 20 PCs were used in the present study as was done previously by others (https: ://www.nealelab.is/uk-biobank/ukbround2announcement).

OSM 2: OLINK PROTEOMICS

Proteomic analysis was performed on 54,306 plasma samples collected from individual UK Biobank participant visits as part of the UK Biobank Pharma Proteomics Project (UKB-PPP) using the Olink® Explore 1536 Proteomics platform, accounting for about 12% of the overall UK Biobank study. This platform uses Proximity Extension Assay (PEA) technology to measure 1,472 protein analytes, including 1,463 unique proteins from inflammation, cancer, cardiometabolic, and neurological panels (3). Sun et al. (4) present additional information, which is discussed below. Several previous studies have also been conducted to assess the repeatability of Olink and other platforms (e.g., (5)).

The assay

Plasma samples were serially diluted to 1:10, 1:100, and 1:1000 before being analyzed in four 384-well plates containing four abundance blocks for each of the four distinct panels per 96 samples. After incubating plasma samples with proximity probes overnight at 4°C, oligonucleotides in close proximity were extended and amplified with DNA polymerase. This phase produces a DNA sequence that was amplified using polymerase chain reaction (PCR 1), resulting in amplicons carrying protein assay data. The total amplicons obtained from each sample's four abundance categories per panel are combined, yielding one well of amplicons per sample. After adding index plates appropriate to each panel to sample plates, a second PCR procedure (PCR 2) was performed to integrate samples from each plate into a single library for each panel. The libraries were then bead purified and quality checked using a Bioanalyzer. Samples were sequenced using S4 flow cells v1.5 on a Novaseq600 (35 cycles), and sequence counts were converted to Normalized Protein eXpression (NPX) values using Olink's MyData Cloud Software.

Olink quality controls

Quality control is built into the Olink workflow. Each sample and abundance block includes three spike-in designed internal controls. The incubation control (Inc Ctrl), a green fluorescent protein (GFP), was utilized to ensure data quality. Data normalization was achieved via extension controls. Amplification controls (Amp Ctrl), which use a synthetic double-stranded DNA template, were used for monitoring and quality control during the PCR step of the procedure. External controls are also available on each plate. To compute the assay limit of detection (LOD), each plate had three negative controls, and a pooled plasma sample was run three times as a plate control sample. A pooled sample control was also performed in duplicate to determine run precision. Each of the four panels for quality control (QC) and correlation analysis contained three unique proteins: IL6, IL8 (CXCL8), and TNF. The QC assessment was evaluated during both run and sample QC. Olink's standard methodologies were used to convert data

into NPX, the log-2 scale relative quantification unit. This procedure entails normalizing matched counts from an assay to the extension control that has been spiked into each sample, then log-2 transforming the results and modifying the amount using the plate control.

NPX calculation and normalization

Samples were drawn from two distinct sets: Set 1 (UKB) and Set 2 (COVID), with samples randomized on plates within each set. The log2 ratio of assay counts in each sample to extension control numbers was used to calculate NPX. The median value for the plate control assay was then removed. This was used to standardize the NPX values for both sets. To adjust for within-batch effects, the assay-specific plate median NPX value was subtracted from set 1 samples, and the batch-specific median NPX value for each assay was added. During this stage of the procedure, each batch's data was normalized. The adjustment factors were then computed by subtracting the assay-specific median NPX value of each batch from the reference batch (batch 1). Following that, additional correction factors were applied to the NPX values of each batch in Set 1. Set 1 was then normalised for both within-batch and across-batch intensity normalization. Set 2 samples were standardized using reference samples that were common to both sets. Plates containing at least one sample from set 2 were randomly assigned a sample from set one. Samples (n=93) from batches 1-6 of set 1 were chosen to represent the dynamic range of NPX values, with a 10% missing rate. These specific samples were then run on the 93 empty wells in batch 7 of set 2. The assay-specific median of the pairwise differences between sets 1 and 2 was then used to calculate specific corrections. Additional factors were used to fine-tune the set of 2 NPX values. The final set of NPX values had both intensity normalized NPX values from set 1 and reference normalized NPX values from set 2.

Data pre-processing and quality checking

The initial UKB-Olink dataset comprised 58,699 samples from 54,309 individuals. After eliminating participant samples that had withdrawn from the study or samples that had not been processed, there were 54,306 participants and 58,362 samples. Following that, samples were deleted due to quality control failures (including missing NPX values), leaving 58,360 samples from 54,304 individuals. Outliers were found using two methods: principal component analysis (PCA) and comparing the median and interquartile range (IQR) of NPX values across proteins by sample. Data points were moved if the PC1 or PC2 values were more than 5 standard deviations (SD) from the mean, the median NPX was more than 5 SDs from the mean of the median, or the IQR of the NPX was more than 5 SDs from the mean of the IQR. Following the removal of outliers, data points that had QC or assay warnings were removed. As a result, the dataset includes 58,240 samples and 54,189 individuals. The intra-individual coefficient of variation for each protein was calculated with duplicate samples and ranged between 2.4% and 25%. Three distinct proteins (CXCL8, IL6, and TNF) were examined across all four panels (cardiometabolic, inflammation, neurology, and oncology). CXCL8 had a mean correlation of 0.96, IL6 had a correlation of 0.92, and TNF had a correlation of 0.81 in all four panels. Batch and plate effects were also explored, but no evidence of either was discovered.

Other sources:(6, 7, 8)

OSM 3: Four-way decomposition models

From 2006 to 2021, every plasma proteome biomarker was identified as a possible mediator or moderator in the association between AD PRS and dementia risk. When a mediator with which the exposure could interact was present, the total effect of the AD PRS exposure on dementia risk was divided into four categories: (i) neither mediation nor interaction; (ii) interaction alone (and not mediation); (iii) both mediation and interaction; and

(iv) only mediation (but no interaction). This recently proposed Stata approach contains strategies for assessing mediation and assigning effects to interactions, allowing for the estimation of the four-way decomposition with parametric or semi-parametric regression models. Importantly, using Cox PH models for the outcome and OLS for each mediator/moderator, up to 1,463 plasma proteomic mediators were identified as alternative possible mediators/moderators. Using the Med4way command (9), we were able to examine mediation and interaction effects for each plasma proteins in an attempt to partition the total effect of AD PRS exposure on the all-cause dementia outcome. In the four-way decomposition model, exogenous factors included age, squared-age, sex, and the top 20 genetic main components. The type I error for each analysis was set at 0.05.

Other sources: *(6, 7, 8)*

OSM 4: **Principal components analysis (PCA) of selected consistent mediating proteins**

PCA is a linear dimensionality reduction technique with applications in data analysis, visualization, and preprocessing. This is accomplished by linearly transforming the data to a new coordinate system (principal components), which allows the directions that capture the most variance in the data to be easily identified. Many studies use the first two major components to plot data in two dimensions and visually highlight clusters of data points that are closely related.

Following the selection process for consistent mediators, each of the 11 consistent mediating proteins ($PROT_{cons\_med}$) was entered into a principal components analysis (PCA) model as measured variables (10), and a number of common principal components were extracted based on common variance, component loadings estimated, and residual variance labeled as uniqueness for each $PROT_{cons\_med}$. The PCA model may be summarized as follows:

$$PROT_{cons\_medi} = \sum_{j=1}^{k} \lambda_{ij} * PCj + \phi_i$$

Where $PROT_{cons\_medi}$ is the standardized z-score for each selected Log2 transformed protein deemed to be consistent mediators (k=11), λ_{ij} is the component loading for each $PROT_{cons\_medi}$ and each component PC_j, PC_j is the standardized z-score for each component j, and φ_i is the residual error, the squared value of which determines uniqueness. The sum of squared component loadings for each $PROT_{\_cons\_medi}$ represents the communality, or common variance, accounted for by the extracted variables.

The eigenvalue>1 rule, often known as the Kaiser rule, was applied, and the scree plot was used to identify the appropriate number of extracted elements for the best model fit. The component loadings were then rotated using varimax orthogonal rotation, and the components were interpreted and labeled as needed, with an arbitrary cutoff point of 0.20 or higher for significant loading that would distinguish between the PC. The component scores (z-scores) were expected to reduce the 11 variables to the main PC, which is likewise measured on a standardized z-score scale. Prediction is made using the regression approach.

Other sources: *(6, 7, 8)*

Supplementary Table 4. Varimax rotated two-factor solution of $PROT_{cons\_medi}$, using 11 selected consistent mediating proteins as measured variables.

	Component loadings, λ_{ij}			Uniqueness, φ_i
	PC1	PC2	PC3	
TNC	-0.030	-0.026	+0.75*	0.35
PVR	+0.277*	+0.170	-0.10	0.76

PILRB	+0.152	+0.168	+0.22*	0.79
NCS1	+0.113	+0.508*	-0.25	0.48
NEFL	-0.139	+0.564*	+0.085	0.42
LDLR	+0.500*	+0.042	-0.22	0.46
KYNU	+0.518*	-0.051	+0.030	0.50
GFAP	-0.098	+0.446*	+0.096	0.63
FURIN	+0.551*	-0.089	+0.139	0.39
DCBLD2	+0.073	+0.073	+0.378*	0.71
BRK1	+0.173	+0.105	+0.469*	0.60
Eigenvalue	2.35	1.53	1.01	
% var explained	21.0	14.0	9.0	

Note: See list of abbreviations.

*Component loading>0.20 in absolute value. Principal components were labeled as follows: PC1: LDLR, KYNU, FURIN, PVR, PC2:NCS1, NEFL, GFAP, PC3: TNC, DCBLD2 and BRK1 based on the combination of significantly high factor loadings and the corresponding measured variables or $PROT_{cons\_medi}$.

Detailed results

Principal component analyses (PCA) were performed on the top 11 significant proteomic mediators that belonged to group C (i.e., consistent mediators), with number of components restricted using the Kaiser rule (eigenvalue>1) (details provided in **OSM4** and **supplementary Table 4, Appendix II**). As such, three principal components were extracted (PC1, PC2 and PC3) (**supplementary Figure 2**, **Appendix VI**). PC1 reflected variance in 4 of top 11 mediators (PVR, LDLR, KYNU and FURIN) with component loadings>0.20, whereas PC2 reflected variance in 3 mediators (NCS1, NfL and GFAP) and PC3 incorporating variances in 4 mediators (TNC, PILRB, DCBLD2 and BRK1). When we entered PC1 into a four-way decomposition model for AD PRS vs. dementia, this potentially mediating principal component explained only 0.3% of the AD PRS-dementia effect (95% CI: 0.02%-0.50%) as PIE, reducing dementia risk by 10% with a standardized effect size with AD PRS estimated at b=-0.020. In contrast, INTREF explained about 2.0% of this TE (P=0.028). Conversely, PC2, which included NfL and GFAP among measured variables with >0.20 component loadings, explained around 4.9% of the TE of AD PRS on dementia risk, as PIE (P<0.001), with an overall percent mediated estimated at 5.9% (P<0.001). More importantly, INTREF explained around 10.7% of TE, with a % eliminated from the TE being around 19.2%, resulting in the attenuated difference between the TE (HR=2.1) and CDE (HR=1.84). As for the third PC (PC3), covering four top mediators, 1.3% of TE was explained by the PIE; 2.0% by INTREF and the percentage eliminated amounted to 4.5%. Some sex differences were noted, particularly with respect to PC3, for which 6% of the TE was eliminated among women and a much smaller proportion was eliminated among men (2.5%). The findings in the overall sample and stratified by sex are presented in **Appendix VII (supplementary Figure 3)** and its associated datasheet and Output available on https://github.com/baydounm/UKB-paper12-supplementarydata. When examining the PCA scores of plasma proteins obtained from the 2019 IGAP AD PRS, the four-way decomposition of the new AD PRS – dementia TE yielded comparable findings as well for PC2 which loaded strongly on GFAP and NfL among others, with less mediation or moderation observed through PC1 or PC3 [**Appendix V** (**supplementary**

datasheet 1) and full Output in https://github.com/baydounm/UKB-paper12-supplementarydata]. OLINK insight and STRING pathway detailed analysis is presented in **OSM 7** (**Appendix II**), as well as visualized in **Appendix VIII** (**supplemental Figure 4**) and **datasheet 2**. Gene ontology (GO) pathways are stored in https://github.com/baydounm/UKB-paper12-supplementarydata.

OSM 5: Updated 2022 AD PRS and PRS pipeline

A polygenic risk score, which is derived by summing the effects of numerous common variations linked to a condition, evaluates an individual's genetic risk for a given disease or trait(11). This genetic risk is increasingly being included as a covariate in statistical analysis due to the growing availability of genetic data in large cohort studies like the UK Biobank(11). In the past, this needed specialized knowledge, but with advancements in technology and data availability, statisticians and epidemiologists may now more easily construct existing scores for use in analyses(11). There are plenty of educational materials available for creating new polygenic risk scores and performing genome-wide association studies, but there aren't as many for just calculating and using pre-existing genetic scores(11). Using the UK Biobank imputed data as a model data set, a new guide outlines the essential steps in this process: selecting appropriate polygenic risk scores from the literature, extracting pertinent genetic variants and confirming their quality, calculating the risk score and important considerations for its inclusion in statistical models(11).

While many of the methods in this tutorial are applicable to other datasets, the authors of the tutorial highlighted several special methods that are necessary when utilizing data in the forms that UK Biobank has chosen(11). This includes some of the difficulties encountered when working with a large number of variants, since some tools' computation times become unfeasible(11). The sheer number of tools available and the challenge for a beginner to evaluate their suitability present a barrier to dealing with genetic data, even though the tutorial has only concentrated on a few that might not be the best for every particular aspect of the process(11). The tutorial was intended to make polygenic risk scores more accessible to more researchers by going into great detail on a few tools that are sufficient for the calculation even at a larger scale(11).

In this manuscript, we applied the same pipeline that was detailed in the tutorial using the GWAS results from a 2022 *Nature Genetics* published paper(12). In short, the European Alzheimer & Dementia Biobank (EADB) consortium has compiled a dataset of 20,464 clinically diagnosed AD cases and 22,244 controls from 15 European countries. The results were meta-analyzed using a proxy-AD GWAS from the UK Biobank dataset(12). The EADB stage I GWAS meta-analysis included 39,106 cases, 46,828 proxy-AD cases, 401,577 controls, and 21,101,114 variants(12). A genome-wide association study involving 111,326 AD cases and 677,663 controls identified 75 risk loci, 42 of which were new(12). Pathway enrichment confirmed amyloid/tau pathways and microglia involvement, with 31 genes identified in relation to novel processes(12). A new genetic risk score was created, resulting in a 1.6- to 1.9-fold increase in AD risk, adjusting for age and the APOE e4 allele(12). The tutorial was run using a Jupyter notebook that was run on a secure server and the imputation genomic files provided for all available UK Biobank participants, particularly those for whom genomic principal components were computed(11) (See **OSM 1**). After running the jupyter notebook on the GWAS results, using 0.5% as the rare variant cutoff, and 50% for palindromic allele frequency, the final AD PRS was based on 39 SNPs which were presented in Table 1 (combining stage I and stage II results) which were then weighted according to effect size(11). The AD PRS was then converted to a z-score similar to the 2019 IGAP AD PRS score(2), using the largest possible sample of UK Biobank(11). The secondary analysis in this paper was carried out on the 2022 AD PRS, mainly the four-way decomposition models for the top plasma proteins uncovered by the IGAP 2019 AD PRS initial analysis (volcano plot) and the consistent mediator principal components analysis. In this secondary analysis the total effect of the new AD PRS on dementia risk was tested through those top proteins and consistent mediating PCs. Replication of previous findings was the focus on this secondary analysis.

In UK Biobank, IMPUTE4.0 was utilized to carry out genotype imputation(1). The most recent IMPUTE4 precompiled binaries can be found at https://jmarchini.org/software/(1). The source code for the BGEN library can be found at https://bitbucket.org/gavinband/bgen(1).

OSM 6: Supplementary literature review on selected consistent protein mediators and their relationship with AD PRS and dementia traits

Supplementary Table 5. DESCRIPTION OF TOP 11 HITS FOR PROTEOMIC MEDIATORS BETWEEN AD PRS AND DEMENTIA

Symbol	Protein/Gene name	Function	Recent papers with relevance to dementia or AD
TNC	Tenascin C	It is implicated in guidance of migrating neurons as well as axons during development, synaptic plasticity, and neuronal regeneration. [provided by RefSeq, Jul 2011]	(13, 14, 15, 16, 17, 18)
PVR	PVR cell adhesion molecule	The protein encoded by this gene is a transmembrane glycoprotein belonging to the immunoglobulin superfamily. The gene serves as a cellular receptor for poliovirus in the first step of poliovirus replication. [provided by RefSeq, Oct 2008]	N/A
PILRB	Paired immunoglobulin like type 2 receptor beta	This gene encodes the activating member of the receptor pair and contains a truncated cytoplasmic tail relative to its inhibitory counterpart (PILRA), that has a long cytoplasmic tail with immunoreceptor tyrosine-based inhibitory (ITIM) motifs. [provided by RefSeq, Jun 2013]	(19, 20)
NCS1	Neuronal calcium sensor 1	The protein encoded by this gene regulates G protein-coupled receptor phosphorylation in a calcium-dependent manner and can substitute for calmodulin. The protein is associated with secretory granules and modulates synaptic transmission and synaptic plasticity. [provided by RefSeq, Jul 2008]	(21)
NEFL	Neurofilament light chain	Neurofilaments functionally maintain the neuronal caliber. They may also play a role in intracellular transport to axons and dendrites. This gene encodes the light chain neurofilament protein. [provided by RefSeq, Oct 2008]	(22, 23, 24, 25, 26, 27)
LDLR	Low density lipoprotein receptor	The low density lipoprotein receptor (LDLR) gene family consists of cell surface proteins involved in	

		receptor-mediated endocytosis of specific ligands.[provided by RefSeq, May 2022]	(28, 29, 30, 31, 32, 33, 34, 35, 36, 37)
KYNU	Kynureninase	Kynureninase is involved in the biosynthesis of NAD cofactors from tryptophan through the kynurenine pathway. [provided by RefSeq, Nov 2010]	(38, 39, 40)
GFAP	Glial fibrillary acidic protein	This gene encodes one of the major intermediate filament proteins of mature astrocytes. It is used as a marker to distinguish astrocytes from other glial cells during development. [provided by RefSeq, Oct 2008]	(26, 41, 42, 43, 44, 45, 46, 47, 48, 49, 50, 51, 52)
FURIN	Furin, paired basic amino acid cleaving enzyme	This gene encodes a member of the subtilisin-like proprotein convertase family, which includes proteases that process protein and peptide precursors trafficking through regulated or constitutive branches of the secretory pathway. It encodes a type 1 membrane bound protease that is expressed in many tissues, including neuroendocrine, liver, gut, and brain. The encoded protein undergoes an initial autocatalytic processing event in the ER and then sorts to the trans-Golgi network through endosomes where a second autocatalytic event takes place and the catalytic activity is acquired. Like other members of this convertase family, the product of this gene specifically cleaves substrates at single or paired basic residues. It is thought to be one of the proteases responsible for the activation of HIV envelope glycoproteins gp160 and gp140, and may play a role in tumor progression. [provided by RefSeq, Aug 2020]	(53, 54, 55, 56)
DCBLD2	Discoidin, CUB, and LCCL domain containing 2	Involved in negative regulation of cell growth and wound healing. Located in cell surface. Is integral component of plasma membrane. [provided by Alliance of Genome Resources, Apr 2022]	(57)
BRK1	BRCA1 DNA repair associated	This gene encodes a 190 kD nuclear phosphoprotein that plays a role in maintaining genomic stability, and it also acts as a tumor suppressor. The BRCA1 gene contains 22 exons spanning about 110 kb of DNA. The encoded protein combines with other tumor suppressors, DNA damage sensors, and signal transducers to form a large multi-subunit protein complex known as the BRCA1-associated genome	N/A

	surveillance complex (BASC). This gene product associates with RNA polymerase II, and through the C-terminal domain, also interacts with histone deacetylase complexes. This protein thus plays a role in transcription, DNA repair of double-stranded breaks, and recombination. [provided by RefSeq, May 2020]	

Protein abbreviations are found at https://www.ncbi.nlm.nih.gov/gene/. *PubMed alternative reference ID in the absence of PMID. Some articles have both, we listed PMIDs in that case.

Protein abbreviations are found at https://www.ncbi.nlm.nih.gov/gene/.

OSM 7: OLINK insight and STRING pathways analyses: detailed methods and results

Detailed methods:

Four-way decomposition of the proteome (i.e., k=1,463 proteins) was carried out, from which plasma proteins with statistically significant TE and PIE (type I error 0.05) were selected. These findings were entered into OLINK insight pathways browser (https://insight.olink.com/) to identify the most often used paths by those mediators (https://github.com/baydounm/UKB-paper12-supplementarydata), which were then displayed as independent and connected pathways. Furthermore, key mediators with statistically significant PIE were analyzed using the Search Tool for the Retrieval of Interacting Genes/Proteins (STRING) (https://string-db.org) and k-means clustering with up to ten clusters. This provided additional information on other proteomic biomarkers identified as pure mediators using PIE, as well as their clustering or functional relationships with top mediators. Within these clusters, gene ontology (GO) pathways were characterized, and the most prominent pathways were identified by combining the lowest false discovery rate with strength value >1. The degree of support or confidence in the functional association between various genes or words based on biological evidence is reflected in the strength of GO.

Other sources: *(6, 7, 8)*

Detailed results:

OLINK insight pathway analysis yielded a total of about 437 pathways, including all statistically significant mediators [k=127 proteins, **Appendix VIII** (**supplemental Figure 4**)]. The immune system, signal transduction, metabolism, disease (including infectious and metabolic disorders), protein metabolism, hemostasis, and the neural system were among the many domains that were implicated. Comprehensive paths are provided in **Appendix IX** (**supplementary datasheet 2**) (p=437). All of the additional datasheets, detailed code, and pertinent outcome datasets that were used to produce the tables and figures are available at the following link: https://github.com/baydounm/UKB-paper12-supplementarydata.

Based on STRING analysis, NEFL and GFAP were both in the same largest cluster of 44 plasma protein mediators (cluster # 9, **supplementary Figure 4**). Among consistent mediators, PVR and FURIN were included in this cluster. Other inconsistent top mediators were also found in this larger cluster, most notably TREM2, SMPD1, SIGLEC1, PLA2G10, IL1RN, CD74 and CCL3. When all mediators were considered, the largest plasma protein clusters contained four of eleven top consistent mediators that were entered into the PCA, along with seven of twenty-nine inconsistent mediators (See KEY_MEDIATORS.txt for full mediator list; **Appendix X**). Gene ontology (GO) and KEGG analyses are also provided for cluster #9 and stored in the github repository: https://github.com/baydounm/UKB-paper12-supplementarydata. More specifically, this largest cluster reflected numerous interconnected biological GO pathways with the lowest FDR coupled with the highest strength ascribed to “response to cytokine” pathway (GO:0034097). The strongest molecular pathway for this cluster with the lowest FDR was “Death receptor activity”: (GO:0005035). A detailed literature review of selected consistent mediator plasma proteins in terms of their association with dementia traits and AD PRS or APOE genotype is presented in **OSM6** and **supplementary Tabel 5**. **Appendix XI** (**Supplementary Figure 5**) visualizes key findings.

OSM 8: SECONDARY ANALYSES

8.1. AD PRS vs. APOE4 carrier status

Among the final selected sample in our analysis, 33,890 UKB participants had data on the APOE genotype. Of these 8,979 were found to be ε4 carriers ($APOE4^+$). Among the $APOE4^+$ group, the main AD PRS score (IGAP-2019) mean±SE was estimated at 1.18±0.010 vs. -0.33±0.004 for $APOE4^-$ (t-test=160, d.f.=33,888, P<0.001), reflecting a strong influence of APOE on this AD PRS. In contrast, for the new AD PRS (2022), although the relationship was in the same direction, there was a weaker relationship whereby $APOE4^+$ had an estimated mean±SE of AD PRS=+0.037±0.010 as compared to the $APOE4^-$ group with mean±SE of -0.0001±0.006. Thus, there was a marked lower influence of APOE genotype and specifically APOE4 status on the new AD PRS compared with the main AD PRS used in this study.

8.2. AD PRS (main and new) vs. all-cause dementia and AD incidence: comparison

With respect to AD PRS's association with all-cause dementia, the association was significantly stronger for the main AD PRS which incorporated the APOE SNPs. Specifically, for all-cause dementia, per SD, the HR=1.80, 95% CI: 1.72-1.88 for the main AD PRS (IGAP-2019) while being markedly weaker for the new AD PRS (2022) that did not incorporate APOE SNPs: Per SD, HR=1.22, 95% CI: 1.15-1.28, P<0.001. For AD incidence, the results were as follows: For the main AD PRS (IGAP 2019): HR=2.19, 95% CI: 2.05-2.33, P<0.001; for the new AD PRS (2022): HR=1.37, 95% CI: 1.26-1.49.

8.3. Main findings for AD incidence outcome, four-way decomposition

8.3.1. Main AD PRS, 86 plasma protein mediators and AD

Appendix XII (**Supplementary datasheet 3**) displays the results of four-way decomposition models that are similar to the ones displayed in **Appendix V** (**Supplementary datasheet 1**) and **Figure S3**, but replacing all-cause dementia with the incidence of AD as the outcome of interest. For the main AD PRS, among 86 plasma protein mediators, most were deemed not significant mediators, while only 3 were inconsistent mediators and 7 were consistent mediators. The latter group was in common with the consistent mediators detected for all-cause dementia analysis in **datasheet 1** but were specific to AD incidence.

8.3.2. Main AD PRS, PCA mediators and AD

Using the PCA results for the consistent mediator for all-cause dementia, a four-way decomposition was carried for the AD incidence outcome: AD PRS → mediator1/2/3 → AD. The results indicated, that mediator 2 was the main PCA component that resulted in 16% proportion eliminated of the TE that was eliminated, most being ascribed to the interaction reference component. This proportion eliminated was reduced to <5% for the remaining two PCA components. This is comparable to the all-cause dementia findings (**Figure S3**).

8.3.3. New AD PRS, 86 plasma protein mediators and AD

Using the AD PRS (2022) as the exposure, coupled with AD incidence as the outcome, the four-way decomposition results across the top 86 plasma proteins are also displayed in **Appendix XII** (**Supplementary datasheet 3**). There were 6 consistent mediators that replicated the findings for all-cause dementia as opposed to the 4 that were detected for all-cause dementia. GFAP and NEFL were in common for the two outcomes and had the strongest proportion mediated, and proportion interaction reference. Specifically, the new AD PRS → GFAP → AD, showed that 35% of

the TE was ascribed to interaction reference, while the new AD PRS → NEFL → AD, showed that 7% of the TE was due to interaction reference. These proportions were comparable to the ones found for all-cause dementia.

8.3.4. New AD PRS, PCA mediators and AD

The PCA findings were also very comparable between AD (**supplementary datasheet 3**) and all-cause dementia incidence (**supplementary datasheet 1**) as outcome. Specifically, mediator 2 was the main PCA component to explain a large proportion of the TE mainly by interaction reference but also by pure indirect effect and mediated interaction. The proportion eliminated by this mediator/moderator in the relationship between the new AD PRS and AD incidence was ~23%, a comparable proportion to the one observed for all-cause dementia (**supplementary datasheet 1**, 21%).

SUPPLEMENTARY REFERENCES

1. Bycroft C, Freeman C, Petkova D, Band G, Elliott LT, Sharp K, et al. The UK Biobank resource with deep phenotyping and genomic data. Nature. 2018;562(7726):203-9.
2. Kunkle BW, Grenier-Boley B, Sims R, Bis JC, Damotte V, Naj AC, et al. Genetic meta-analysis of diagnosed Alzheimer's disease identifies new risk loci and implicates Abeta, tau, immunity and lipid processing. Nat Genet. 2019;51(3):414-30.
3. Wik L, Nordberg N, Broberg J, Bjorkesten J, Assarsson E, Henriksson S, et al. Proximity Extension Assay in Combination with Next-Generation Sequencing for High-throughput Proteome-wide Analysis. Mol Cell Proteomics. 2021;20:100168.
4. Sun BB, Chiou J, Traylor M, Benner C, Hsu YH, Richardson TG, et al. Plasma proteomic associations with genetics and health in the UK Biobank. Nature. 2023;622(7982):329-38.
5. Petrera A, von Toerne C, Behler J, Huth C, Thorand B, Hilgendorff A, et al. Multiplatform Approach for Plasma Proteomics: Complementarity of Olink Proximity Extension Assay Technology to Mass Spectrometry-Based Protein Profiling. J Proteome Res. 2021;20(1):751-62.
6. Beydoun HA, Beydoun MA, Noren Hooten N, Weiss J, Li Z, Georgescu MF, et al. Mediating and moderating effects of plasma proteomic biomarkers on the association between poor oral health problems and incident dementia: The UK Biobank study. Geroscience. 2024;46(5):5343-63.
7. Beydoun MA, Beydoun HA, Hu YH, Li Z, Georgescu MF, Noren Hooten N, et al. Mediating and moderating effects of plasma proteomic biomarkers on the association between poor oral health problems and brain white matter microstructural integrity: the UK Biobank study. Mol Psychiatry. 2024.
8. Beydoun MA, Beydoun HA, Noren Hooten N, Meirelles O, Li Z, El-Hajj ZW, et al. Hospital-treated prevalent infections, the plasma proteome and incident dementia among UK older adults. iScience. 2023;26(12):108526.
9. Discacciati A, Bellavia A, Lee JJ, Mazumdar M, Valeri L. Med4way: a Stata command to investigate mediating and interactive mechanisms using the four-way effect decomposition. Int J Epidemiol. 2018.
10. Sharma S. Applied multivariate techniques. USA: Wiley; 1996.
11. Collister JA, Liu X, Clifton L. Calculating Polygenic Risk Scores (PRS) in UK Biobank: A Practical Guide for Epidemiologists. Front Genet. 2022;13:818574.
12. Bellenguez C, Kucukali F, Jansen IE, Kleineidam L, Moreno-Grau S, Amin N, et al. New insights into the genetic etiology of Alzheimer's disease and related dementias. Nat Genet. 2022;54(4):412-36.
13. Dong Y, Zhao K, Qin X, Du G, Gao L. The mechanisms of perineuronal net abnormalities in contributing aging and neurological diseases. Ageing Res Rev. 2023;92:102092.
14. Hasanzadeh Z, Nourazarian A, Nikanfar M, Laghousi D, Vatankhah AM, Sadrirad S. Evaluation of the Serum Dkk-1, Tenascin-C, Oxidative Stress Markers Levels and Wnt Signaling Pathway Genes Expression in Patients with Alzheimer's Disease. J Mol Neurosci. 2021;71(4):879-87.
15. Jayakumar AR, Apeksha A, Norenberg MD. Role of Matricellular Proteins in Disorders of the Central Nervous System. Neurochem Res. 2017;42(3):858-75.
16. Minta K, Portelius E, Janelidze S, Hansson O, Zetterberg H, Blennow K, et al. Cerebrospinal Fluid Concentrations of Extracellular Matrix Proteins in Alzheimer's Disease. J Alzheimers Dis. 2019;69(4):1213-20.
17. Morawski M, Bruckner MK, Riederer P, Bruckner G, Arendt T. Perineuronal nets potentially protect against oxidative stress. Exp Neurol. 2004;188(2):309-15.
18. Xie K, Liu Y, Hao W, Walter S, Penke B, Hartmann T, et al. Tenascin-C deficiency ameliorates Alzheimer's disease-related pathology in mice. Neurobiol Aging. 2013;34(10):2389-98.

19. Ryan KJ, White CC, Patel K, Xu J, Olah M, Replogle JM, et al. A human microglia-like cellular model for assessing the effects of neurodegenerative disease gene variants. Sci Transl Med. 2017;9(421).
20. Karch CM, Ezerskiy LA, Bertelsen S, Alzheimer's Disease Genetics C, Goate AM. Alzheimer's Disease Risk Polymorphisms Regulate Gene Expression in the ZCWPW1 and the CELF1 Loci. PLoS One. 2016;11(2):e0148717.
21. Franco R, Aguinaga D, Reyes I, Canela EI, Lillo J, Tarutani A, et al. N-Methyl-D-Aspartate Receptor Link to the MAP Kinase Pathway in Cortical and Hippocampal Neurons and Microglia Is Dependent on Calcium Sensors and Is Blocked by alpha-Synuclein, Tau, and Phospho-Tau in Non-transgenic and Transgenic APP(Sw,Ind) Mice. Front Mol Neurosci. 2018;11:273.
22. Sainio MT, Rasila T, Molchanova SM, Jarvilehto J, Torregrosa-Munumer R, Harjuhaahto S, et al. Neurofilament Light Regulates Axon Caliber, Synaptic Activity, and Organelle Trafficking in Cultured Human Motor Neurons. Front Cell Dev Biol. 2021;9:820105.
23. Kamalian A, Ho SG, Patel M, Lewis A, Bakker A, Albert M, et al. Exploratory Assessment of Proteomic Network Changes in Cerebrospinal Fluid of Mild Cognitive Impairment Patients: A Pilot Study. Biomolecules. 2023;13(7).
24. Kulsirichawaroj P, Suksangkharn Y, Nam DE, Pho-Iam T, Limwongse C, Chung KW, et al. Gene Distribution in Pediatric-Onset Inherited Peripheral Neuropathy: A Single Tertiary Center in Thailand. J Neuromuscul Dis. 2024;11(1):191-9.
25. Fernandez-Martos CM, King AE, Atkinson RA, Woodhouse A, Vickers JC. Neurofilament light gene deletion exacerbates amyloid, dystrophic neurite, and synaptic pathology in the APP/PS1 transgenic model of Alzheimer's disease. Neurobiol Aging. 2015;36(10):2757-67.
26. Guo Y, You J, Zhang Y, Liu WS, Huang YY, Zhang YR, et al. Plasma proteomic profiles predict future dementia in healthy adults. Nat Aging. 2024;4(2):247-60.
27. Mirza Z, Rajeh N. Identification of Electrophysiological Changes in Alzheimer's Disease: A Microarray Based Transcriptomics and Molecular Pathway Analysis Study. CNS Neurol Disord Drug Targets. 2017;16(9):1027-38.
28. Masliah E, Spencer B. Applications of ApoB LDLR-Binding Domain Approach for the Development of CNS-Penetrating Peptides for Alzheimer's Disease. Methods Mol Biol. 2015;1324:331-7.
29. de Oliveira FF, Chen ES, Smith MC, Bertolucci PHF. Selected LDLR and APOE Polymorphisms Affect Cognitive and Functional Response to Lipophilic Statins in Alzheimer's Disease. J Mol Neurosci. 2020;70(10):1574-88.
30. Guven G, Vurgun E, Bilgic B, Hanagasi H, Gurvit H, Ozer E, et al. Association between selected cholesterol-related gene polymorphisms and Alzheimer's disease in a Turkish cohort. Mol Biol Rep. 2019;46(2):1701-7.
31. Krishnan N, Chen X, Donnelly-Roberts D, Mohler EG, Holtzman DM, Gopalakrishnan SM. Small Molecule Phenotypic Screen Identifies Novel Regulators of LDLR Expression. ACS Chem Biol. 2020;15(12):3262-74.
32. Perkovic R, Francic M, Petrovic R, Ozretic D, Skara S, Pecin I, et al. Early-onset Alzheimer's disease due to novel LDLR gene mutation. Acta Neurol Belg. 2024;124(1):325-7.
33. Yao L, Gu X, Song Q, Wang X, Huang M, Hu M, et al. Nanoformulated alpha-mangostin ameliorates Alzheimer's disease neuropathology by elevating LDLR expression and accelerating amyloid-beta clearance. J Control Release. 2016;226:1-14.
34. Whelan CD, Mattsson N, Nagle MW, Vijayaraghavan S, Hyde C, Janelidze S, et al. Multiplex proteomics identifies novel CSF and plasma biomarkers of early Alzheimer's disease. Acta Neuropathol Commun. 2019;7(1):169.

35. Riad A, Lengyel-Zhand Z, Zeng C, Weng CC, Lee VM, Trojanowski JQ, et al. The Sigma-2 Receptor/TMEM97, PGRMC1, and LDL Receptor Complex Are Responsible for the Cellular Uptake of Abeta42 and Its Protein Aggregates. Mol Neurobiol. 2020;57(9):3803-13.
36. Akyol O, Akyol S, Chou MC, Chen S, Liu CK, Selek S, et al. Lipids and lipoproteins may play a role in the neuropathology of Alzheimer's disease. Front Neurosci. 2023;17:1275932.
37. Rivas-Dominguez A, Mohamed-Mohamed H, Jimenez-Palomares M, Garcia-Morales V, Martinez-Lopez L, Orta ML, et al. Metabolic Disturbance of High-Saturated Fatty Acid Diet in Cognitive Preservation. Int J Mol Sci. 2023;24(9).
38. Sutphin GL, Backer G, Sheehan S, Bean S, Corban C, Liu T, et al. Caenorhabditis elegans orthologs of human genes differentially expressed with age are enriched for determinants of longevity. Aging Cell. 2017;16(4):672-82.
39. Cheng D, Qin ZS, Zheng Y, Xie JY, Liang SS, Zhang JL, et al. Minocycline, a classic antibiotic, exerts psychotropic effects by normalizing microglial neuroinflammation-evoked tryptophan-kynurenine pathway dysregulation in chronically stressed male mice. Brain Behav Immun. 2023;107:305-18.
40. Majewski M, Kozlowska A, Thoene M, Lepiarczyk E, Grzegorzewski WJ. Overview of the role of vitamins and minerals on the kynurenine pathway in health and disease. J Physiol Pharmacol. 2016;67(1):3-19.
41. Gogishvili D, Illes-Toth E, Harris MJ, Hopley C, Teunissen CE, Abeln S. Structural flexibility and heterogeneity of recombinant human glial fibrillary acidic protein (GFAP). Proteins. 2023.
42. Gonzales MM, Vela G, Philip V, Trevino H, LaRoche A, Wang CP, et al. Demographic and Clinical Characteristics Associated With Serum GFAP Levels in an Ethnically Diverse Cohort. Neurology. 2023;101(15):e1531-e41.
43. Keski-Pukkila M, Karr JE, Posti JP, Berghem K, Kotilainen AK, Blennow K, et al. Preliminary Evaluation of the Scandinavian Guidelines for Initial Management of Minimal, Mild, and Moderate Head Injuries with Glial Fibrillary Acidic Protein. Neurotrauma Rep. 2024;5(1):50-60.
44. Rodriguez JJ, Gardenal E, Zallo F, Arrue A, Cabot J, Busquets X. Astrocyte S100beta expression and selective differentiation to GFAP and GS in the entorhinal cortex during ageing in the 3xTg-Alzheimer's disease mouse model. Acta Histochem. 2024;126(1):152131.
45. Sanchez E, Wilkinson T, Coughlan G, Mirza S, Baril AA, Ramirez J, et al. Association of plasma biomarkers with cognition, cognitive decline, and daily function across and within neurodegenerative diseases: Results from the Ontario Neurodegenerative Disease Research Initiative. Alzheimers Dement. 2023.
46. Suchy-Dicey AM, Longstreth WT, Jr., Rhoads K, Umans J, Buchwald D, Grabowski T, et al. Plasma biomarkers of Alzheimer's disease and related dementias in American Indians: The Strong Heart Study. Alzheimers Dement. 2024.
47. Wojdala AL, Bellomo G, Gaetani L, Toja A, Chipi E, Shan D, et al. Trajectories of CSF and plasma biomarkers across Alzheimer's disease continuum: disease staging by NF-L, p-tau181, and GFAP. Neurobiol Dis. 2023;189:106356.
48. Ally M, Sugarman MA, Zetterberg H, Blennow K, Ashton NJ, Karikari TK, et al. Cross-sectional and longitudinal evaluation of plasma glial fibrillary acidic protein to detect and predict clinical syndromes of Alzheimer's disease. Alzheimers Dement (Amst). 2023;15(4):e12492.
49. Yang Z, Sreenivasan K, Toledano Strom EN, Osse AML, Pasia LG, Cosme CG, et al. Clinical and biological relevance of glial fibrillary acidic protein in Alzheimer's disease. Alzheimers Res Ther. 2023;15(1):190.
50. Fong TG, Vasunilashorn SM, Kivisakk P, Metzger ED, Schmitt EM, Marcantonio ER, et al. Biomarkers of neurodegeneration and neural injury as potential predictors for delirium. Int J Geriatr Psychiatry. 2024;39(1):e6044.

51. Gammie SC, Messing A, Hill MA, Kelm-Nelson CA, Hagemann TL. Large-scale gene expression changes in APP/PSEN1 and GFAP mutation models exhibit high congruence with Alzheimer's disease. PLoS One. 2024;19(1):e0291995.
52. Zheng X, Yang J, Hou Y, Shi X, Liu K. Prediction of clinical progression in nervous system diseases: plasma glial fibrillary acidic protein (GFAP). Eur J Med Res. 2024;29(1):51.
53. Bennett BD, Denis P, Haniu M, Teplow DB, Kahn S, Louis JC, et al. A furin-like convertase mediates propeptide cleavage of BACE, the Alzheimer's beta -secretase. J Biol Chem. 2000;275(48):37712-7.
54. Marcinkiewicz M. BetaAPP and furin mRNA concentrates in immature senile plaques in the brain of Alzheimer patients. J Neuropathol Exp Neurol. 2002;61(9):815-29.
55. Schwab C, Hosokawa M, Akiyama H, McGeer PL. Familial British dementia: colocalization of furin and ABri amyloid. Acta Neuropathol. 2003;106(3):278-84.
56. Ayyubova G, Gychka SG, Nikolaienko SI, Alghenaim FA, Teramoto T, Shults NV, et al. The Role of Furin in the Pathogenesis of COVID-19-Associated Neurological Disorders. Life (Basel). 2024;14(2).
57. Seow WJ, Matsuo K, Hsiung CA, Shiraishi K, Song M, Kim HN, et al. Association between GWAS-identified lung adenocarcinoma susceptibility loci and EGFR mutations in never-smoking Asian women, and comparison with findings from Western populations. Hum Mol Genet. 2017;26(2):454-65.
