# Supplementary figures and images for: Alzheimer’s Disease polygenic risk, the plasma proteome, and dementia incidence among UK older adults
Source: GeroScience. 2024 Nov 26;47(2):2507–23. doi: 10.1007/s11357-024-01413-8 (PMC11978584; doi:10.1007/s11357-024-01413-8)

Supplementary Figure 1. Participant Flowchart

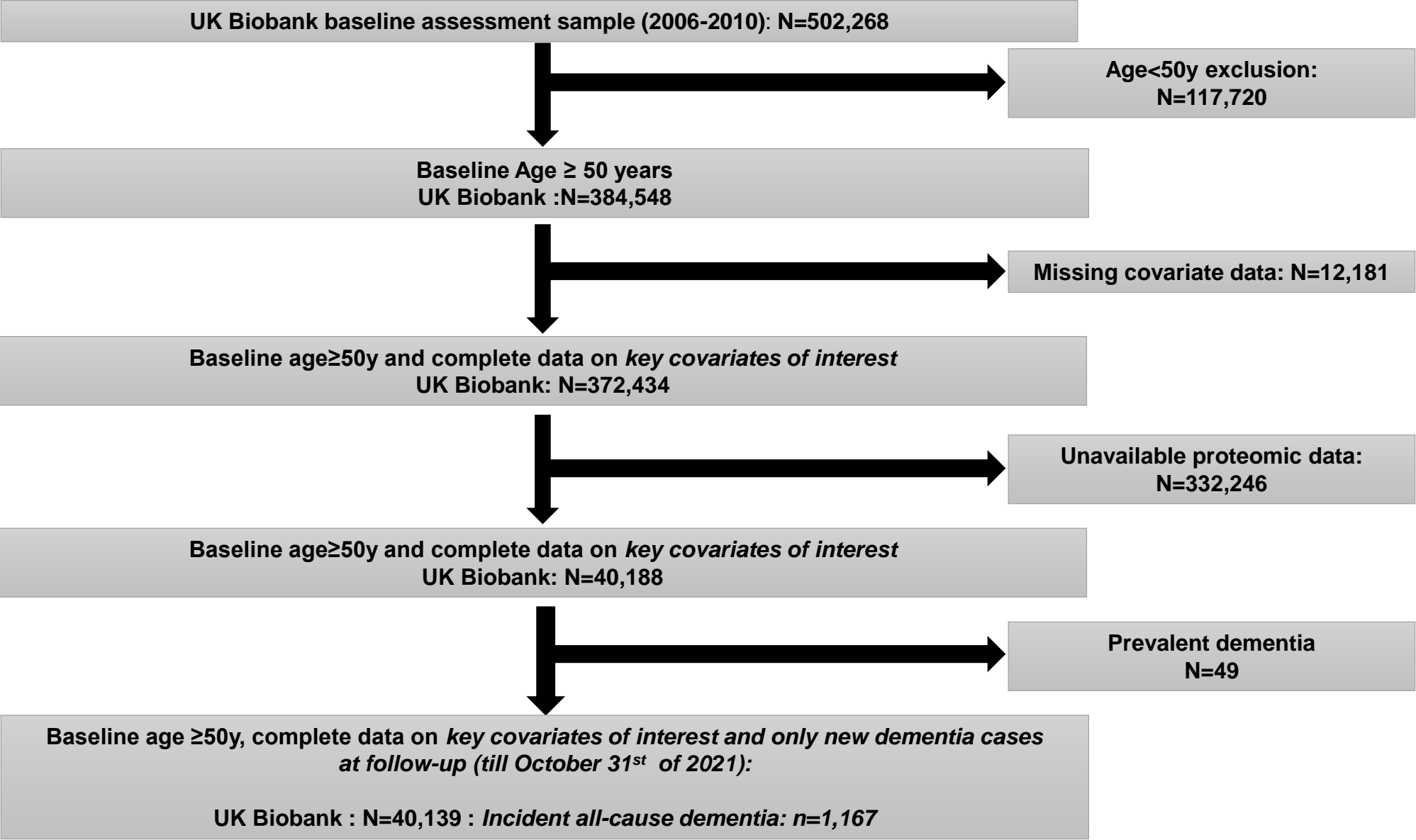

Supplement: Supplementary file 3 — Supplementary file3 Appendix III – Supplementary Figure S1 (PDF 57.7 KB) [file 11357_2024_1413_MOESM3_ESM.pdf]
