# Supplementary material for: Alzheimer’s Disease polygenic risk, the plasma proteome, and dementia incidence among UK older adults
Source: GeroScience. 2024 Nov 26;47(2):2507–23. doi: 10.1007/s11357-024-01413-8 (PMC11978584; doi:10.1007/s11357-024-01413-8)
Supplement: Supplementary file 4 — Supplementary file4 Appendix IV– Four-way decomposition models appendix (PDF 176 KB ) [file 11357_2024_1413_MOESM4_ESM.pdf]

Supplementary Methods File

Contents

1 Four-Way Decomposition Models **1**
1.1 *Background* 1
1.2 *Implementation* 2

2 Inconsistent Mediation **3**

** Please note the text from this file is taken and only slightly modified from the supplementary file our team created for doi: 10.1016/j.isci.2023.108526.[1]

1 Four-Way Decomposition Models

1.1 *Background*

Let Y denote the response, A the exposure (in this case the Alzheimer's Disease polygenic risk score (AD PRS; continuous)), a its realized value, M the mediator (individual plasma proteomic marker levels or principal component scores), m its realized value, and $\boldsymbol{c}$ the vector of confounders. Under the assumption of no confounding, namely:

i. $Y_{am} \coprod A|C$

ii. $Y_{am} \coprod M|(A, C)$

iii. $M_a \coprod A|C$

iv. $Y_{am} \coprod A*|C$

that the effect of A on Y is unconfounded conditional on C (i), that the effect of M on Y is unconfounded conditional on (A, C), (ii), the effect of A on M is unconfounded conditional on C (iii), and that any mediator outcome confounders are not affected by the exposure (iv), we can partition the sources of total effect of the model into four components (Equation 1).[2]

$$TE = CDE + INT_{ref} + INT_{med} + PIE \tag{1}$$

We interpret this model to reflect that the total effect of the exposure, A, on the outcome, Y, is a sum of the *controlled direct effect* (CDE–i.e., the effect of A on Y not due to any interaction or mediation), *the reference interaction* (INT_{ref}–i.e., the effect of interaction only), *the mediated interaction* (INT_{med}–i.e., the effect of interaction and mediation), and *the purely indirect effect* (PIE–i.e., the indirect effect only). Additional details on the four-way decomposition model are provided in an original publication that we refer the readers to.[2]

1.2 *Implementation*

Estimating the components of the partitioned model in Equation 1 involves fitting two regression models and applying the parameter estimates for the final computation of the components. We direct the reviewers to a detailed explanation of the *med4way* command in Stata, while we offer a concise overview of how to apply and estimate the effects.[3] Assuming no unmeasured confounding, we can estimate the four components of the model on a population level, but not on an individual level (as detailed above in **1.1**).Two regression models are provided: one for the expected value of Y given the exposure, mediator, and confounders (Equation 2), and another for the expected value of M given the exposure and confounders (Equation 3).

$$\mathrm{E}[Y|(a, m, \boldsymbol{c})] = \theta_0 + \theta_1 a + \theta_2 m + \theta_3 a * m + \boldsymbol{\theta}_c c \quad (2)$$

$$\mathrm{E}[M|(a, \boldsymbol{c})] = \beta_0 + \beta_1 a + \boldsymbol{\beta}_c^T \boldsymbol{c} \quad (3)$$

Estimates of these parameters therefore facilitate the direct computation of estimates of the four component sources of variation for the total effect (TE):

$$\mathrm{E}[CDE|c] = \theta_1(a - a')$$

$$\mathrm{E}[INT_{ref}|c] = \theta_3(\beta_0 + \beta_1 a' + \boldsymbol{\beta}_c^T \boldsymbol{c})(a - a')$$

$$\mathrm{E}[INT_{med}|c] = \theta_3 \beta_1 (a - a')(a - a')$$

$$\mathrm{E}[PIE|c] = (\theta_2 \beta_1 + \theta_3 \beta_1 a')(a - a')$$

where $a = 1$ and $a' = 0$ if the exposure is binary (as it is in this case).[4] The models we describe are generalizable and *Med4way* can handle outcome variables from several distributions (e.g., binomial, log-binomial, Poisson, negative binomial, Weibull, Cox, etc.).[3] In our analysis, $\mathrm{E}[Y|(a, m, c)]$ and $\mathrm{E}[M|(a, c)]$ are specified as follows:

$$\lambda(t|x, v, \boldsymbol{z}) = \lambda_0(t) + \theta_1 x + \theta_2 v + \theta_3 x * v + \boldsymbol{\theta}_z^T \boldsymbol{z} \quad (4)$$

$$\mathrm{E}[V|(x, \boldsymbol{z})] = \beta_0 + \beta_1 x + \boldsymbol{\beta}_z^T \boldsymbol{z} \quad (5)$$

where, in Equation 4, we model the log hazard at time t as a function of x, AD PRS, v, an individual proteomic biomarker or a principal component score, and $\boldsymbol{z}$, the vector of confounders/covariates discussed in the manuscript. In Equation 5, we model the expectation of the mediator, V, as a function of AD PRS and the other covariates using ordinary least squares.

2 Inconsistent Mediation

Inconsistent mediation can be understood as when a direct and indirect effect operate in opposing directions.[5] In cases where the association between X and Y is not significant, a mediated effect may still exist. This can be attributed to the mediator explaining part or all of the relationship through its causal path. Goldberg et al. (1996) conducted a study investigating the impact of an anabolic steroid prevention program on high school football players.[6] The program, aimed at several mediators, resulted in a notable decrease in intentions to use steroids. The mediator's role in the use of anabolic and androgenic steroids was discovered to have an inconsistent influence on mediation. The investigation revealed that the total impact ($\tau^{\wedge}$ = -0.139) was closer to zero than the direct effect ($\tau^{\wedge'}$ = -0.181), and the third variable ($\alpha\beta$ = 0.042) and direct effects were likewise of opposite sign. This coefficient pattern suggests the existence of uneven mediation or a suppressor effect. The program seems to amplify the justifications for using anabolic steroids, thus resulting in heightened inclinations to take steroids. Nevertheless, there were additional substantial mediation effects linked to the intervention that decreased the inclination to use steroids. The study emphasizes the significance of taking into account the mediational approach to avoid steroid use among high school football players. Researchers examined the "Black-White depression paradox" by analyzing data from the National Epidemiologic Survey on Alcohol and Related Conditions III conducted in the United States from 2012 to 2013.[7] The authors suggested two hypotheses to explain the connection between racial group membership, exposure to life stressors, and major depressive disorder (MDD): an effect modification model and an inconsistent mediator model. Evidence of inconsistent mediation indicated a more complicated association between racial group membership and MDD.

References

1. Beydoun, M. A. *et al.* Hospital-treated prevalent infections, the plasma proteome and incident dementia among UK older adults. *iScience* **26** (2023).
2. VanderWeele, T. J. A unification of mediation and interaction: a four-way decomposition. *Epidemiology (Cambridge, Mass.)* **25,** 749 (2014).

3. Discacciati, A., Bellavia, A., Lee, J. J., Mazumdar, M. & Valeri, L. *Med4way: a Stata command to investigate mediating and interactive mechanisms using the four-way effect decomposition* 2019.

4. VanderWeele, T. J. & Tchetgen Tchetgen, E. J. Mediation analysis with time varying exposures and mediators. *Journal of the Royal Statistical Society Series B: Statistical Methodology* **79,** 917–938 (2017).

5. MacKinnon, D. P., Fairchild, A. J. & Fritz, M. S. Mediation analysis. *Annu. Rev. Psychol.* **58,** 593–614 (2007).

6. Goldberg, L. *et al.* Effects of a multidimensional anabolic steroid prevention intervention: The Adolescents Training and Learning to Avoid Steroids (ATLAS) program. *Jama* **276,** 1555–1562 (1996).

7. Pamplin II, J. R., Rudolph, K. E., Keyes, K. M., Susser, E. S. & Bates, L. M. Investigating a Paradox: Towards Better Understanding the Relationships Between Racial Group Membership, Stress, and Major Depressive Disorder. *American Journal of Epidemiology,* 1845–1853 (2023).
