# Supplementary material for: Alzheimer’s Disease polygenic risk, the plasma proteome, and dementia incidence among UK older adults
Source: GeroScience. 2024 Nov 26;47(2):2507–23. doi: 10.1007/s11357-024-01413-8 (PMC11978584; doi:10.1007/s11357-024-01413-8)

FIGURE S2. Results of principal components analysis for top consistent mediators (k=11), overall (N_{max}=34,574): UK biobank 2006-2021

(A) Scree plot

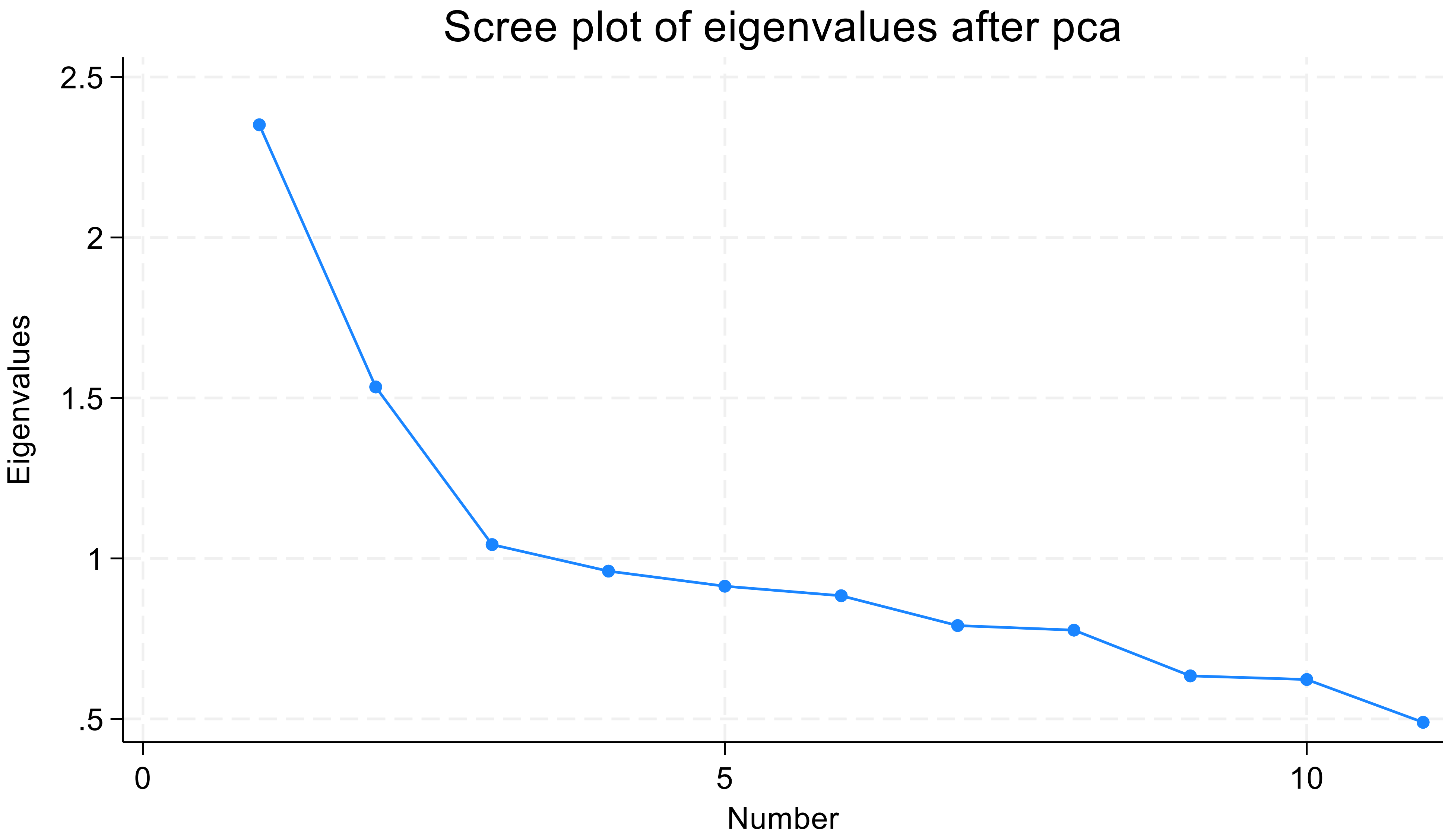

(B) Principal Component loading plot (PC1, PC2 and PC3)

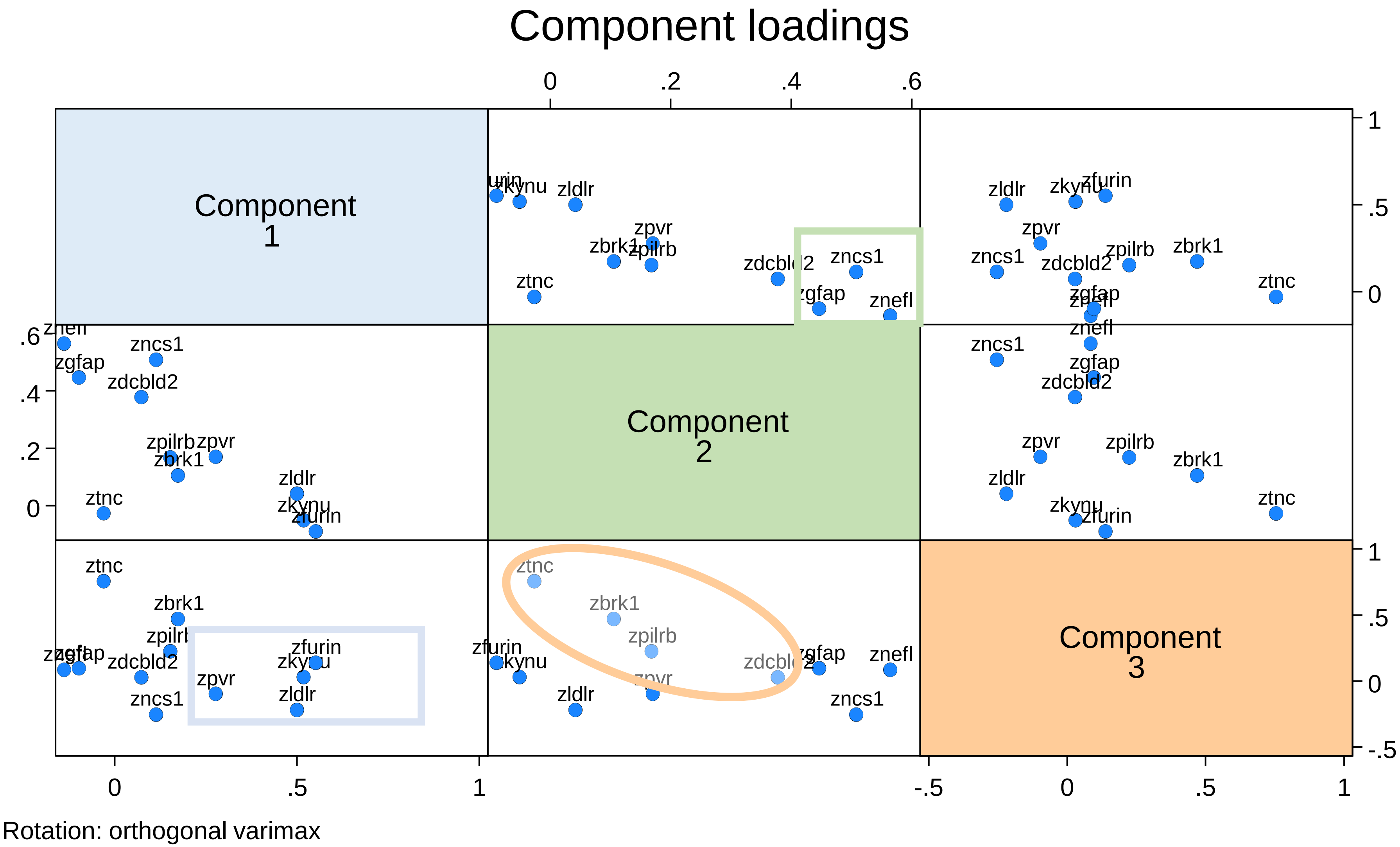

Supplement: Supplementary file 6 — Supplementary file6 Appendix VI – Supplementary Figure S2 (PDF 97.0 KB) [file 11357_2024_1413_MOESM6_ESM.pdf]
