# Supplementary material for: Alzheimer’s Disease polygenic risk, the plasma proteome, and dementia incidence among UK older adults
Source: GeroScience. 2024 Nov 26;47(2):2507–23. doi: 10.1007/s11357-024-01413-8 (PMC11978584; doi:10.1007/s11357-024-01413-8)
Supplement: Supplementary file 7 — Supplementary file7 Appendix VII – Supplementary Figure S3 (PDF 170 KB) [file 11357_2024_1413_MOESM7_ESM.pdf]

FIGURE S3. Four-way decomposition of the association between AD PRS and incidence of all-cause dementia by principal component score of selected plasma proteomic biomarkers with consistent mediation (k=11), overall (N=34,574): UK biobank 2006-2021

Abbreviations: AD=Alzheimer's Disease; ereri_cde= excess relative risk due to neither mediation nor interaction or controlled direct effect;

ereri_intmed= excess relative risk due to mediated interaction or mediated interaction; ereri_intref= excess relative risk due to interaction only or reference interaction;

ereri_pie= excess relative risk due to mediation only or pure indirect effect; p_cde=proportion of total effect that is controlled direct effect;

p_intmed=proportion of total effect that is mediated interaction; p_intref=proportion of total effect that is reference interaction;

p_pie=proportion of total effect that is pure indirect effect; PRS=Polygenic Risk Score; tereri= Total excess relative risk; UK=United Kingdom.

See **supplementary Table 5** for protein abbreviations and **supplementary Table 4** (**Appendix II**) for results of PCA. Other Protein abbreviations are found at https://www.ncbi.nlm.nih.gov/gene/.

*P<0.05; **P<0.010; ***P<0.001

(A) Four-way decomposition with PC1

(B) Four-way decomposition with PC2

(C) Four-way decomposition with PC3

(A)

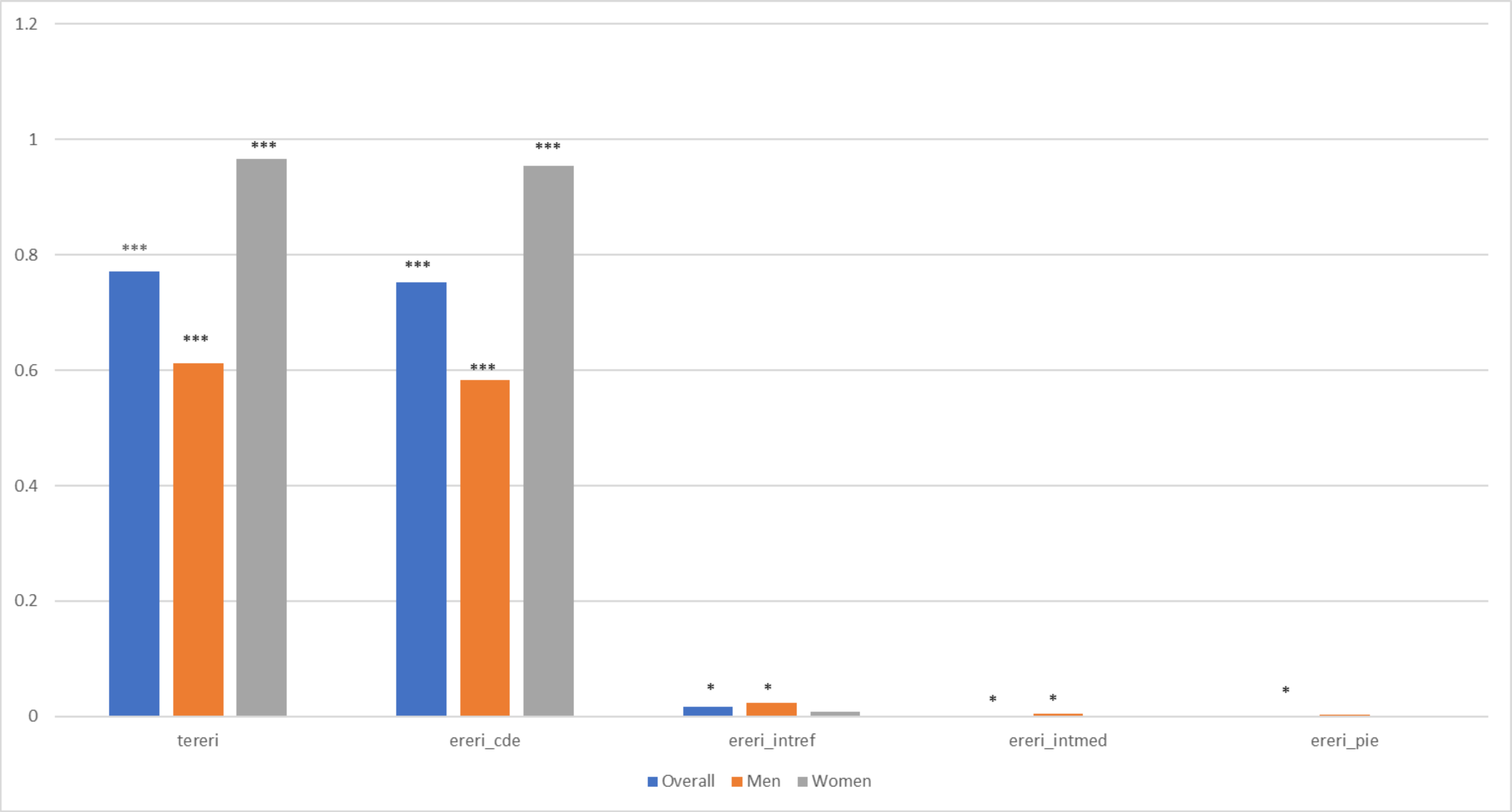

	Overall	Men	Women
tereri	0.772	0.612	0.966
ereri_cde	0.752	0.583	0.955
ereri_intref	0.016	0.023	0.0088
ereri_intmed	0.002	0.004	0.0012
ereri_pie	0.002	0.003	0.0015

*P<0.05; **P<0.010; ***P<0.001

(B)

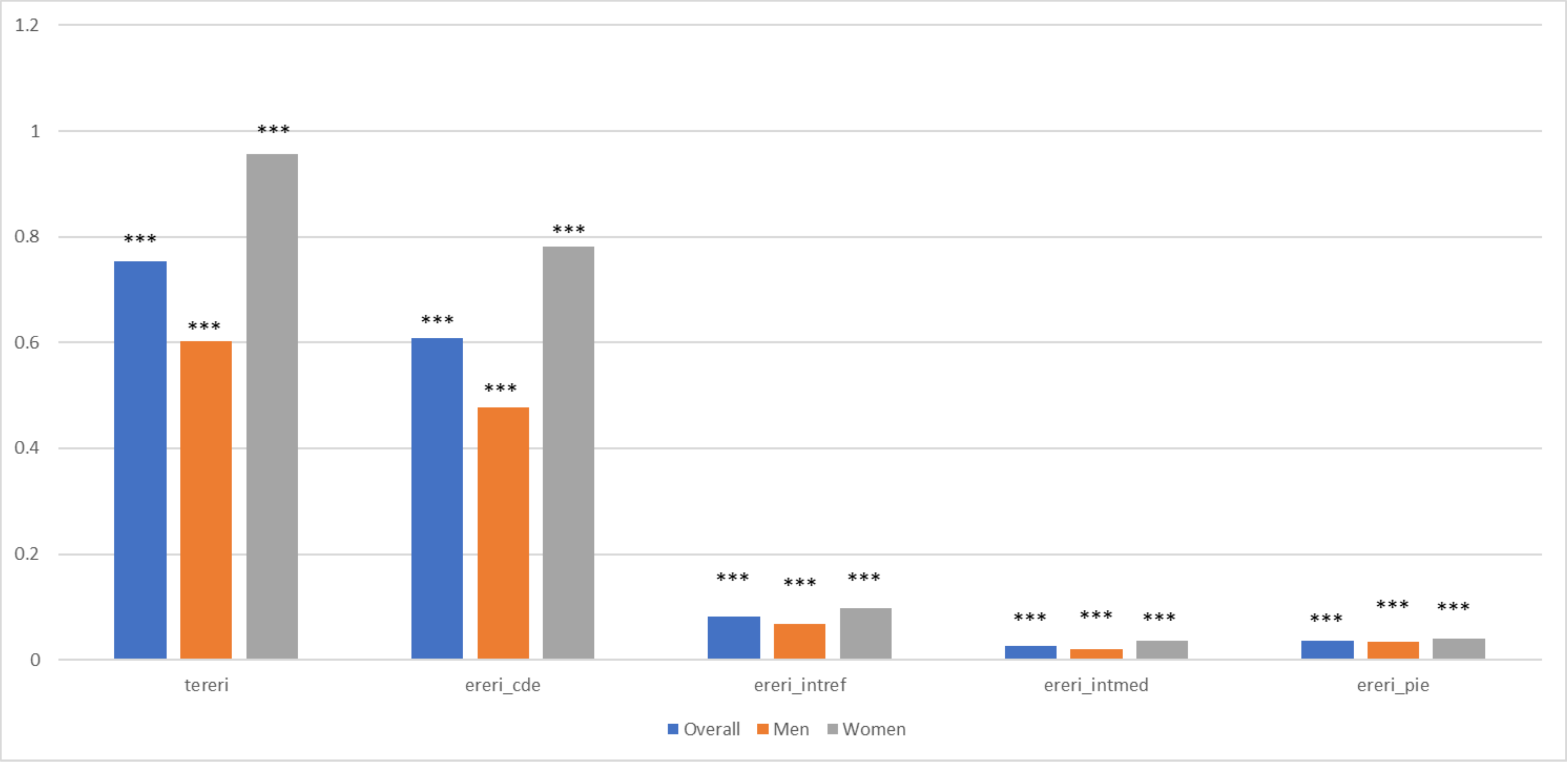

	Overall	Men	Women
tereri	0.754	0.602	0.956
ereri_cde	0.609	0.478	0.782
ereri_intref	0.081	0.069	0.098
ereri_intmed	0.027	0.021	0.036
ereri_pie	0.037	0.034	0.041

*P<0.05; **P<0.010; ***P<0.001

(C)

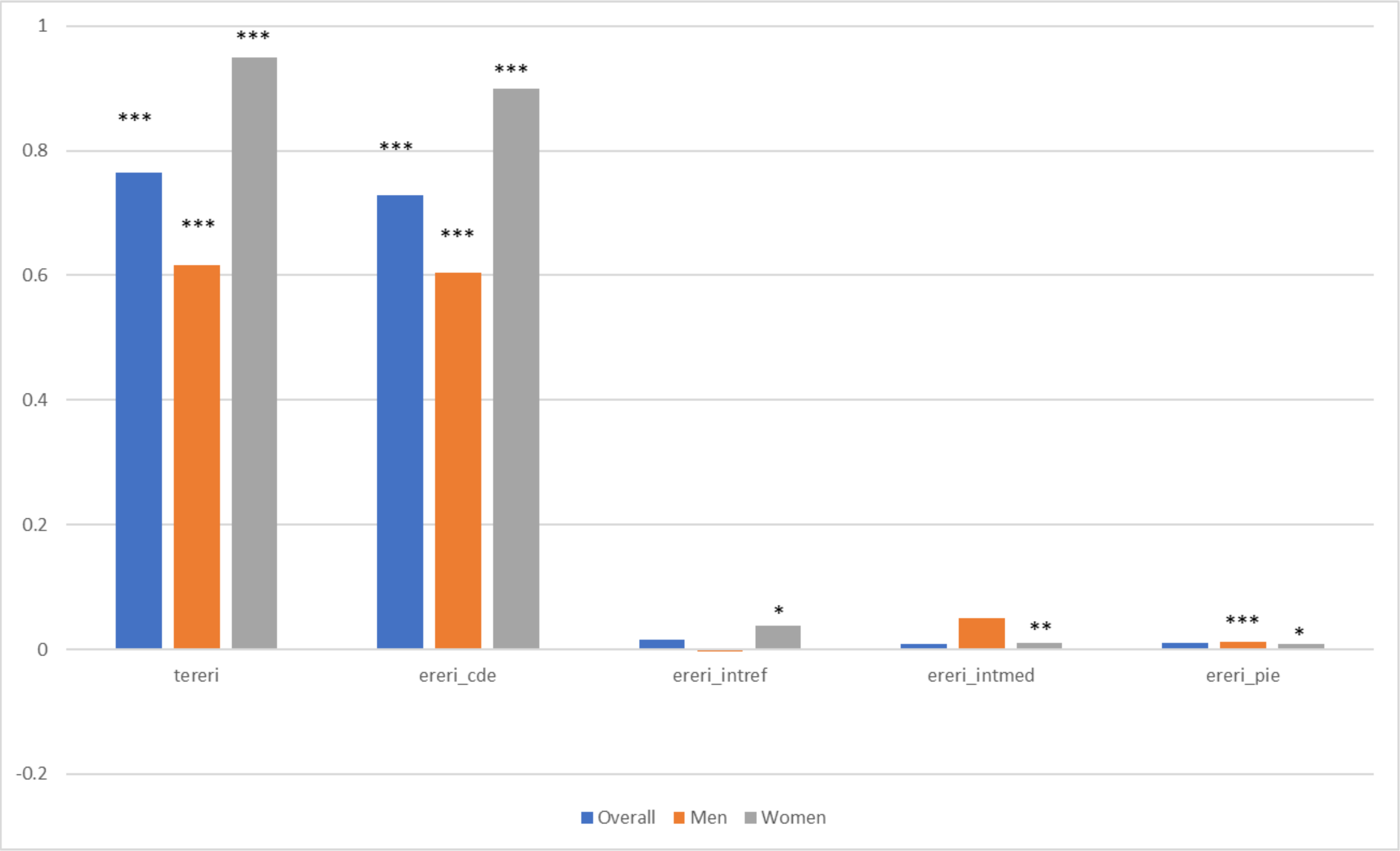

	Overall	Men	Women
tereri	0.764	0.617	0.95
ereri_cde	0.729	0.605	0.90
ereri_intref	0.016	-0.004	0.038
ereri_intmed	0.009	0.05	0.011
ereri_pie	0.01	0.012	0.009

*P<0.05; **P<0.010; ***P<0.001
