# Supplementary material for: Alzheimer’s Disease polygenic risk, the plasma proteome, and dementia incidence among UK older adults
Source: GeroScience. 2024 Nov 26;47(2):2507–23. doi: 10.1007/s11357-024-01413-8 (PMC11978584; doi:10.1007/s11357-024-01413-8)
Supplement: Supplementary file 8 — Supplementary file8 Appendix VIII– Supplementary Figure S4 (PDF 0.98 MB) [file 11357_2024_1413_MOESM8_ESM.pdf]

FIGURE S4. OLINK Insight Pathway browser findings for all plasma proteomic biomarkers found to be significant mediators (PIE, p<0.05) and STRING analysis (k=127 proteins)

Sources: https://insight.olink.com/. The full list of plasma protein biomarkers included into the insight pathway analysis is found on github: https://github.com/baydounm/UKB-paper12-supplementarydata. The same list was included in the STRING analysis: https://string-db.org

Abbreviations: PIE=Pure indirect effect. Protein abbreviations are found at https://www.ncbi.nlm.nih.gov/gene/.

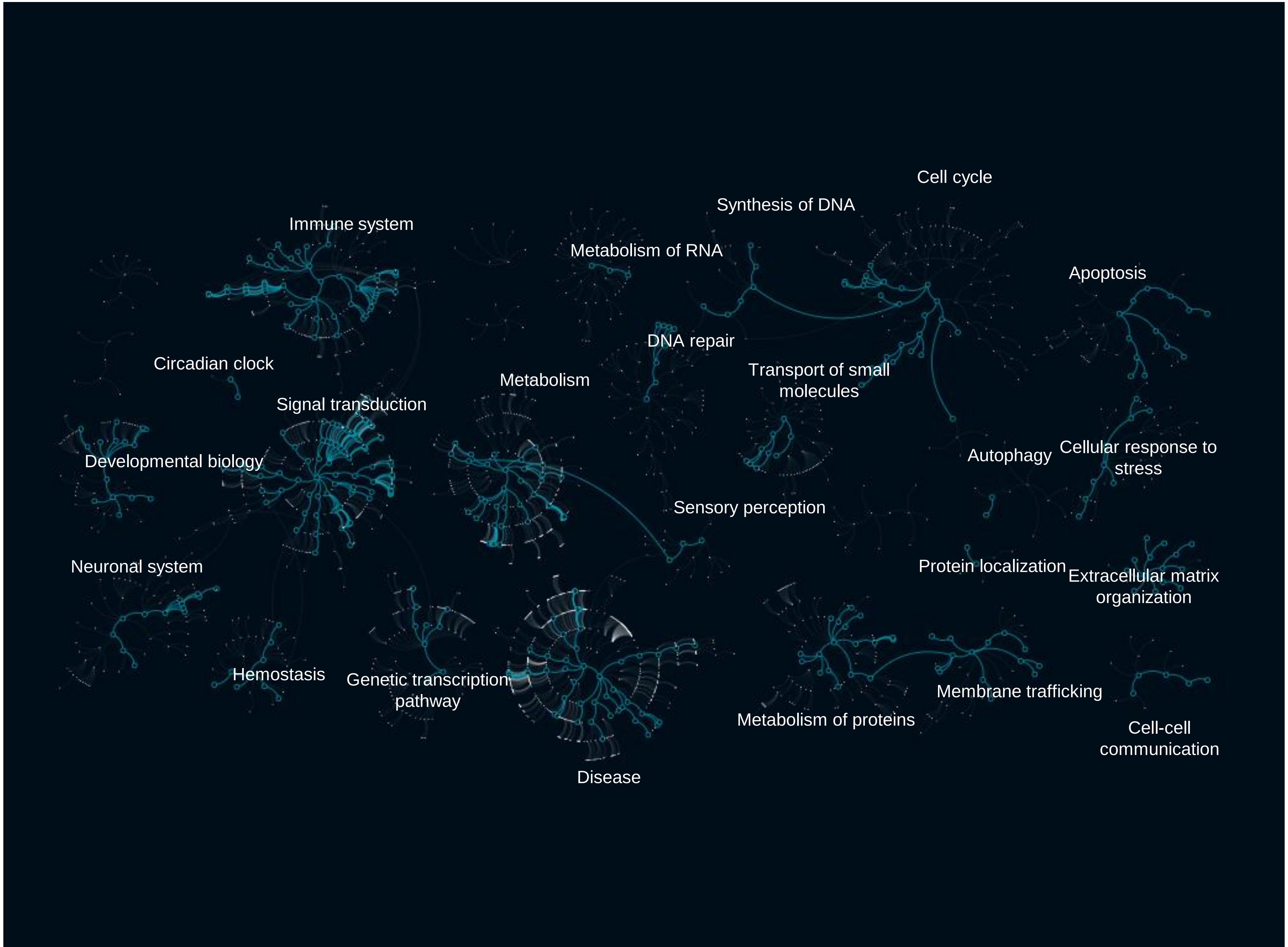
Cell cycle
Synthesis of DNA
Immune system
Metabolism of RNA
Apoptosis
DNA repair
Circadian clock
Transport of small molecules
Metabolism
Signal transduction
Developmental biology
Autophagy
Cellular response to stress
Sensory perception
Neuronal system
Protein localization
Extracellular matrix organization
Hemostasis
Genetic transcription pathway
Membrane trafficking
Metabolism of proteins
Cell-cell communication
Disease

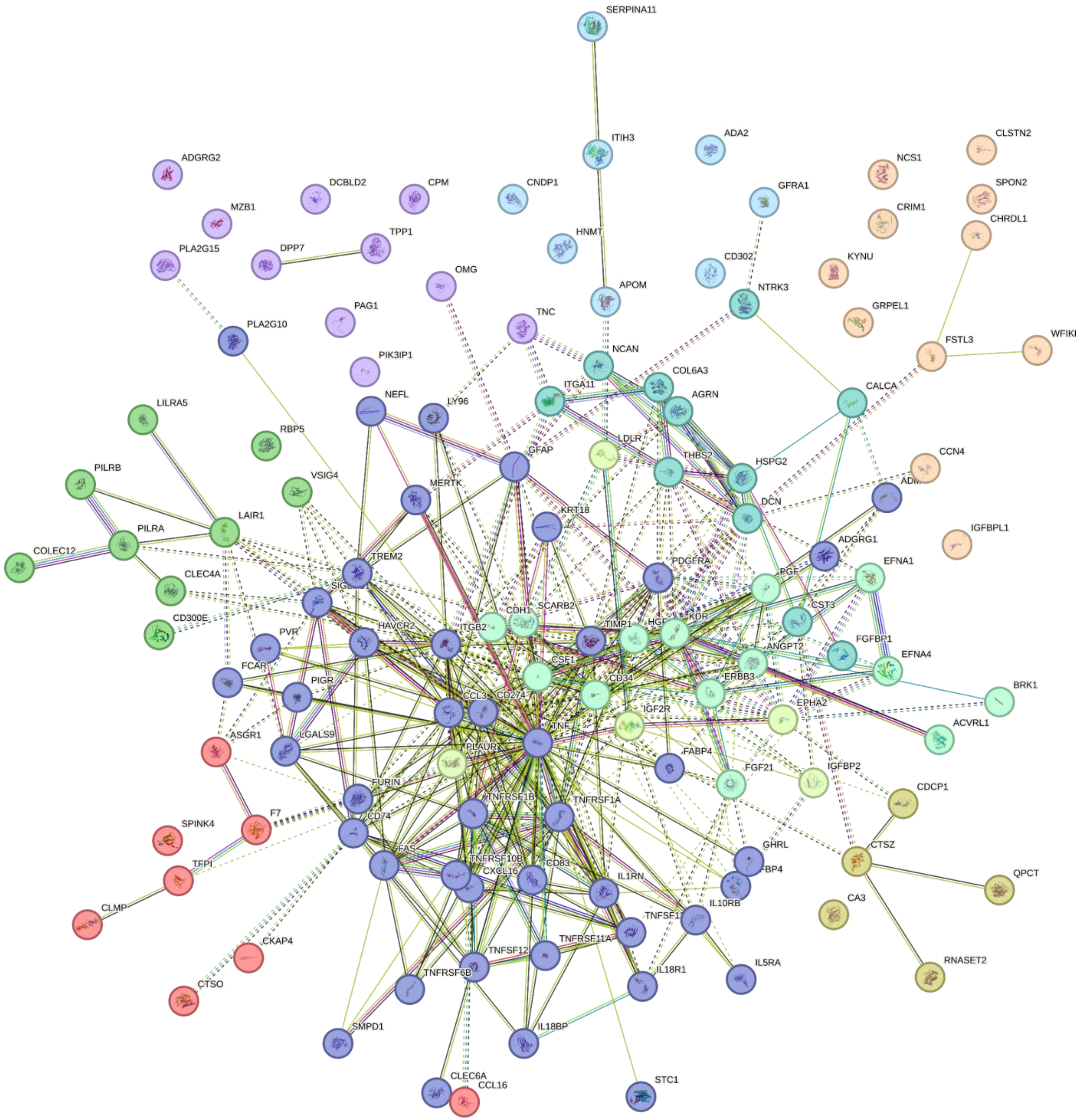

bubble	cluster Id	gene count	protein names
	Cluster 1	8	ASGR1,CCL16,CKAP4,CLMP,CTSO,F7,SPINK4,TFPI
	Cluster 2	11	CCN4,CHRDL1,CLSTN2,CRIM1,FSTL3,GRPEL1,IGFBPL1,KYNU,NCS1,SPON2,WFIKK...
	Cluster 3	5	CA3,CDCP1,CTSZ,QPCT,RNASET2
	Cluster 4	5	EPHA2,IGF2R,IGFBP2,LDLR,PLAUR
	Cluster 5	9	CD300E,CLEC4A,COLEC12,LAIR1,LILRA5,PILRA,PILRB,RBP5,VSIG4
	Cluster 6	14	ACVRL1,ANGPT2,BRK1,CD34,CDH1,CSF1,EFNA1,EFNA4,ERBB3,FGF21,HGF,KDR,PG...
	Cluster 7	11	AGRN,CALCA,COL6A3,CST3,DCN,FGFBP1,HSPG2,ITGA11,NCAN,NTRK3,THBS2
	Cluster 8	8	ADA2,APOM,CD302,CNDP1,GFRA1,HNMT,ITIH3,SERPINA11
	Cluster 9	44	ADGRG1,ADM,CCL3,CD274,CD74,CD83,CLEC6A,CXCL16,FABP4,FAS,FCAR,FURIN,GF...
	Cluster 10	11	ADGRG2,CPM,DCBLD2,DPP7,MZB1,OMG,PAG1,PIK3IP1,PLA2G15,TNC,TPP1

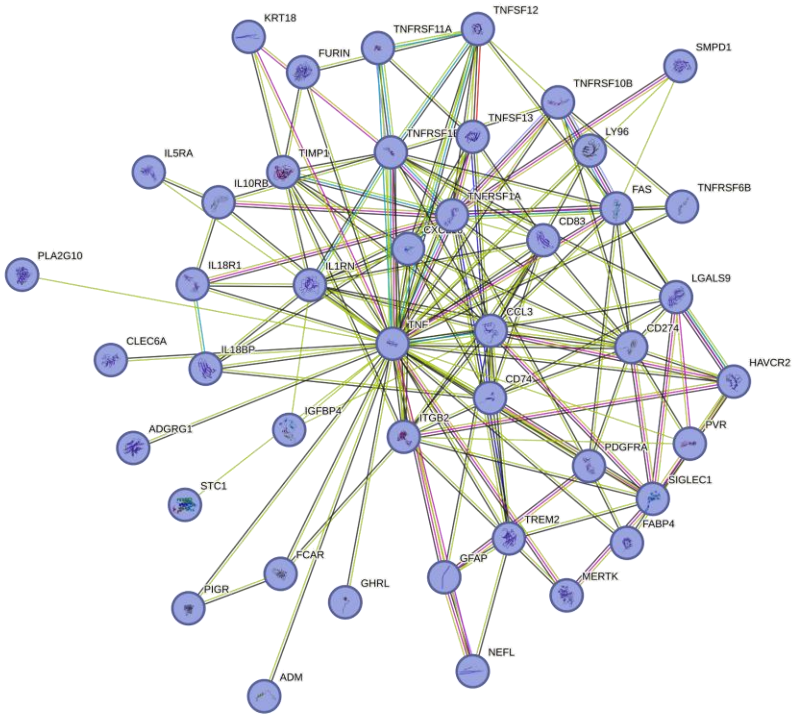

Cluster #9
