# Supplementary material for: Alzheimer’s Disease polygenic risk, the plasma proteome, and dementia incidence among UK older adults
Source: GeroScience. 2024 Nov 26;47(2):2507–23. doi: 10.1007/s11357-024-01413-8 (PMC11978584; doi:10.1007/s11357-024-01413-8)
Supplement: Supplementary file 11 — Supplementary file11 Appendix XI – Supplementary Figure S5 (PDF 586 KB) [file 11357_2024_1413_MOESM11_ESM.pdf]

FIGURE S5. Summary of Findings

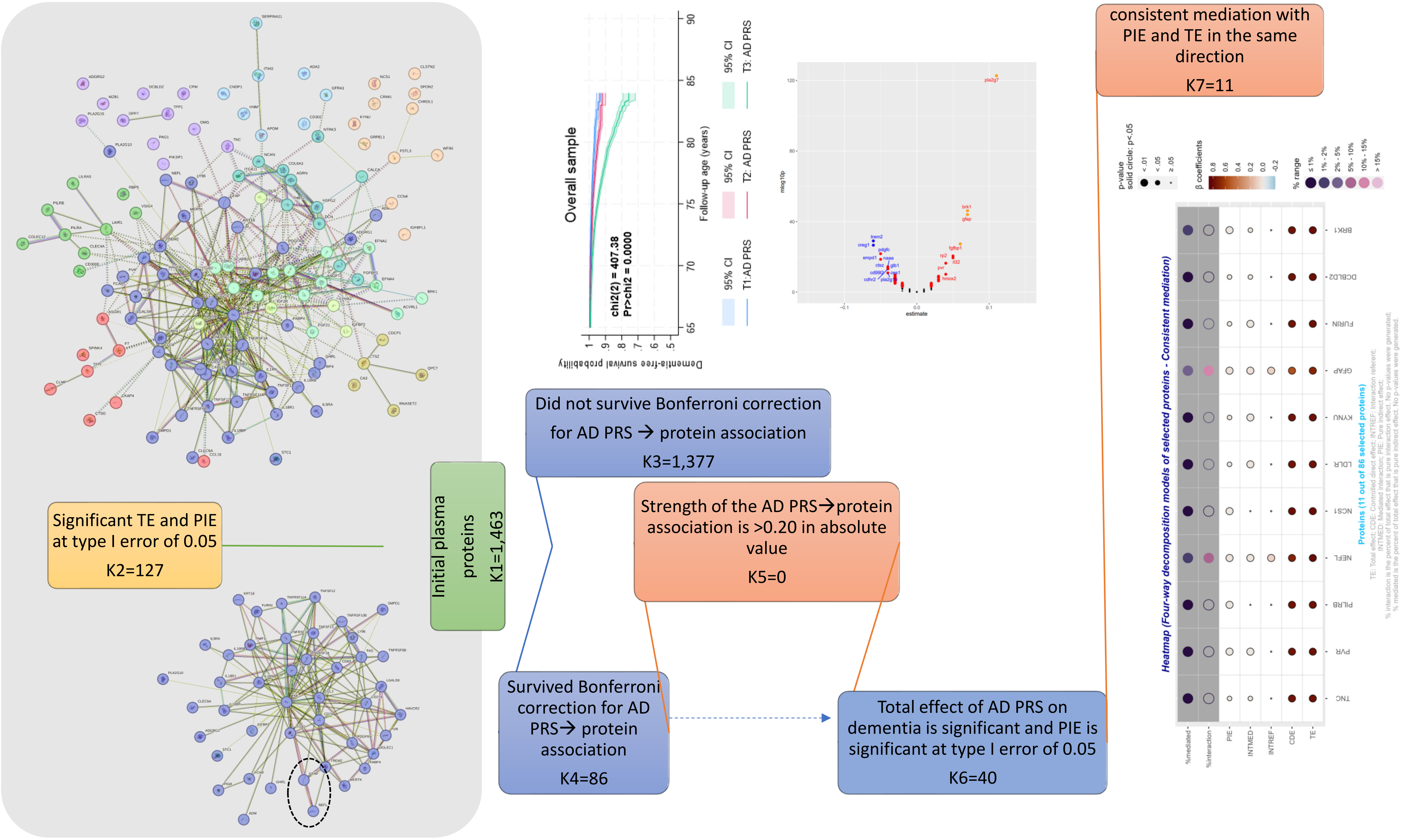
consistent mediation with PIE and TE in the same direction
K7=11
Did not survive Bonferroni correction for AD PRS → protein association
K3=1,377
Strength of the AD PRS→protein association is >0.20 in absolute value
K5=0
Significant TE and PIE at type I error of 0.05
K2=127
Initial plasma proteins
K1=1,463
Survived Bonferroni correction for AD PRS→ protein association
K4=86
Total effect of AD PRS on dementia is significant and PIE is significant at type I error of 0.05
K6=40
Overall sample
chi2(2) = 407.38
Pr>chi2 = 0.0000
Dementia-free survival probability
Follow-up age (years)
T1:AD PRS
T2: AD PRS
T3: AD PRS
95% CI
estimate
Heatmap (Four-way decomposition models of selected proteins - Consistent mediation)
Proteins (11 out of 86 selected proteins)
